# HERV-W group evolutionary history in non-human primates: characterization of ERV-W orthologs in *Catarrhini* and related ERV groups in *Platyrrhini*

**DOI:** 10.1186/s12862-018-1125-1

**Published:** 2018-01-19

**Authors:** Nicole Grandi, Marta Cadeddu, Jonas Blomberg, Jens Mayer, Enzo Tramontano

**Affiliations:** 1 Department of Life and Environmental Sciences, University of Cagliari, Cagliari, Italy; 2 Department of Medical Sciences, Uppsala University, Uppsala, Sweden; 3 Institute of Human Genetics, University of Saarland, Homburg, Germany; 4 Istituto di Ricerca Genetica e Biomedica (IRGB), CNR, Monserrato, Italy

**Keywords:** Comparative genomics, Endogenous retroviruses, HERV-W, Syncytin, ERV1–1, Viral evolution, Monkey and ape retroviruses

## Abstract

**Background:**

The genomes of all vertebrates harbor remnants of ancient retroviral infections, having affected the germ line cells during the last 100 million years. These sequences, named Endogenous Retroviruses (ERVs), have been transmitted to the offspring in a Mendelian way, being relatively stable components of the host genome even long after their exogenous counterparts went extinct. Among human ERVs (HERVs), the HERV-W group is of particular interest for our physiology and pathology. A HERV-W provirus in locus 7q21.2 has been coopted during evolution to exert an essential role in placenta, and the group expression has been tentatively linked to Multiple Sclerosis and other diseases. Following up on a detailed analysis of 213 HERV-W insertions in the human genome, we now investigated the ERV-W group genomic spread within primate lineages.

**Results:**

We analyzed HERV-W orthologous loci in the genome sequences of 12 non-human primate species belonging to *Simiiformes* (parvorders *Catarrhini* and *Platyrrhini*), *Tarsiiformes* and to the most primitive *Prosimians*. Analysis of HERV-W orthologous loci in non-human *Catarrhini* primates revealed species-specific insertions in the genomes of Chimpanzee (3), Gorilla (4), Orangutan (6), Gibbon (2) and especially Rhesus Macaque (66). Such sequences were acquired in a retroviral fashion and, in the majority of cases, by L1-mediated formation of processed pseudogenes. There were also a number of LTR-LTR homologous recombination events that occurred subsequent to separation of *Catarrhini* sub-lineages. Moreover, we retrieved 130 sequences in Marmoset and Squirrel Monkeys (family *Cebidae*, *Platyrrhini* parvorder), identified as ERV1–1_CJa based on RepBase annotations, which appear closely related to the ERV-W group. Such sequences were also identified in *Atelidae* and *Pitheciidae*, representative of the other *Platyrrhini* families. In contrast, no ERV-W-related sequences were found in genome sequence assemblies of *Tarsiiformes* and *Prosimians*.

**Conclusions:**

Overall, our analysis now provides a detailed picture of the ERV-W sequences colonization of the primate lineages genomes, revealing the exact dynamics of ERV-W locus formations as well as novel insights into the evolution and origin of the group.

**Electronic supplementary material:**

The online version of this article (10.1186/s12862-018-1125-1) contains supplementary material, which is available to authorized users.

## Background

The genomes of all vertebrates include a portion of sequences of viral origin, namely Endogenous Retroviruses (ERVs). ERVs belong to Class I Transposable Elements (TEs), representing remnants of ancient infections that occurred mostly during the last 100 million years [1]. An essential step of the retroviral infectious cycle is reverse transcription, in which the single-stranded RNA genome is converted into a double-stranded DNA (provirus) and stably integrated in the host cell genome. In the case of ERVs, such integration occurred in the germ line cells, allowing the subsequent Mendelian inheritance of proviral sequences through the offspring.

If not severely mutated, ERVs share with exogenous retroviruses a typical proviral structure, where two Long Terminal Repeats (LTRs) flank *gag, pro, pol* and *env* genes. Briefly, *gag* encodes matrix, capsid and nucleocapsid proteins; *pro* and *pol* encode the viral enzymes Protease, Reverse Transcriptase, Ribonuclease H and Integrase; and *env* encodes the envelope surface and transmembrane domains. The 5′ and 3′ LTRs are formed during reverse transcription from two unique regions (U3 and U5) separated by a repeated portion (R), and are identical at the time of formation.

ERVs, like all TEs, had a major role in vertebrate evolution [2] and greatly influenced host genomes by providing new functions and evolutionary stimuli, causing relevant physiological effects on the host [3–5]. ERV colonization could cause genetic alterations, insertional mutagenesis, non-homologous recombination, rearrangements and disruption of genes [1, 3, 6–9]. ERV LTRs could provide additional regulatory elements, potentially acting as bidirectional promoters, enhancers, alternative splice and polyadenylation sites [3, 9–17]. Indeed, some ERV LTRs have been coopted as promoters/enhancers of nearby genes involved in embryonic development and pluripotency maintenance that was likely beneficial to the host’s evolution [18]. ERV proteins can likewise being coopted and greatly influence the host’s biology and evolution, as in the case of functional envelope proteins (Env) produced by an ERV-W and an ERV-FRD provirus, Syncytin-1 and Syncytin-2, respectively, that are involved in the placental syncytiotrophoblast formation and in the maternal immune tolerance to the fetus [19–22]. Notably, while Syncytin-1 is conserved in the genomes of Hominoids only and Syncytin-2 is shared by all primates except *Tarsiiformes* and *Prosimians*, functionally similar Env-derived proteins from different ERV groups have been domesticated independently on multiple occasions for the placental functions of several mammalian lineages, thus representing a process of convergent evolution [23, 24]. Also ERV sequences devoid of functional Open Reading Frames (ORFs) can nevertheless modulate important host functions. For instance, spread of ERVs during mammalian evolution dispersed a great number of interferon-inducible enhancers, thus shaping an effective regulatory network of innate immunity [25]. ERVs were also reported to influence the defence systems via RNA transcripts that can modulate host functions in a variety of mechanisms, among which RNA interference and innate immunity sensing of double-stranded RNA [10, 26].

Beside the contributions to (human) physiology and evolution, some pathological roles have also been suggested for HERVs [3–5] and their expression has been tentatively linked to a number of diseases [27–31], although no unequivocal cause-effect relationships have been established so far [3, 31, 32].

**Fig. 1:**
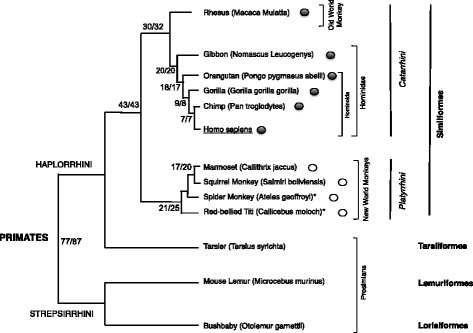
Schematic of the phylogeny of the primate species analyzed in this study. Presence of (H)ERV-W or (H)ERV-W-related sequences in respective species is indicated with a filled or an empty circle, respectively. Primates’ parvorders and infraorders are indicated in italics and bold, respectively. Estimated ages of divergences of evolutionary lineages in millions of years ago are given near tree nodes and were taken from Steiper and Young 2006 [39] (first number) and Perelman et al. 2011 [40] (second number). Species marked with an * lack assembled reference genome sequences

While ERVs and their exogenous counterparts are currently co-existing in some vertebrates [33–35], exogenous retroviruses that formed HERV insertions have gone extinct millions of years ago (MYa), and usually cannot be studied as replicating viruses. However, considerable information on ancestral retroviruses can be obtained from HERV sequences, constituting approximately 8% of the human genomic DNA [36], by comparative analysis of shared (orthologous) elements within non-human primate species. We recently analyzed the human genome sequence assembly GRCh37/hg19 with RetroTector software [37], characterizing ~ 3200 near complete HERV insertions [38]. The most ancient HERV groups formed before the separation of parvorders *Catarrhini* (which includes the families *Cercopithecidae*, also known as Old World Monkeys, OWM, and *Hominoidea*) and *Platyrrhini* (also known as New World Monkeys, NWM), that occurred ~ 40 MYa [39, 40] (Fig. 1), being thus shared between primate species of both parvorders, as in the case of HERV-L and HERV-H) [41]. Many other HERV groups, such as HERV-E and HERV-K(HML-2), are evolutionarily younger and have been acquired after the evolutionary separation of *Catarrhini* and *Platyrrhini*.

Among HERVs, the HERV-W group has recently drawn considerable interest. In fact, as mentioned above, a HERV-W provirus in locus 7q21.2 (ERVWE1) retained an intact ORF producing a functional Env-like protein, Syncytin-1, coopted for placenta morphogenesis and homeostasis [19, 20, 42], while the group’s overall expression has been investigated in various human pathological contexts [43].

In a previous study, we described in detail the distribution and genetic composition of 213 HERV-W loci in the human genome assembly GRCh37/hg19, providing a detailed overview of this HERV group [44]. Briefly, the HERV-W group comprises 65 proviruses, acquired through retroviral replication and having complete 5′ and 3′ LTRs; 135 processed pseudogenes, generated by L1 (Long Interspersed Nuclear Elements 1) retrotransposition and having accordingly truncated LTRs [45, 46]; and 13 unclassifiable elements lacking both LTRs. Phylogenetic and structural analysis classified HERV-W members into subgroups 1 and 2 that were acquired along the *Catarrhini* evolutionary lineage approximately between 40 and 20 MYa, after the lineage’s separation from parvorder *Platyrrhini* [44].

In order to further characterize the HERV-W group throughout primate evolution, we investigated HERV-W homologous sequences in primate species with publicly available genome assemblies (Fig. 1). In particular, we i) analyzed the HERV-W loci non-human orthologs, as well as the additional species-specific ERV-W sequences lacking orthologs in humans, in the genome sequences of 5 *Catarrhini* species, specifically Rhesus Macaque and 4 great apes (Gibbon, Orangutan, Gorilla and Chimpanzee); ii) identified and characterized ERV elements closely related to ERV-W, named ERV1–1 in RepBase, in *Platyrrhini* species Marmoset and Squirrel Monkey (family *Cebidae*); iii) found support for the presence of such ERV-W related elements also in Spider Monkey and Red-bellied Titi species, belonging to the other *Platyrrhini* families (*Atelidae* and *Pitheciidae*, respectively); and iv) corroborated the lack of (H)ERV-W closely related elements in *Tarsiiformes* and in the more primitive *Prosimians* (including *Lemuriformes* and *Lorisiformes*).

Taken together, our findings provide a detailed description of the ERV-W sequences presence and distribution within primate genomes, and further depict the group evolutionary history in various primate lineages. Importantly, comparative analyses allowed us to characterize ERV-W species-specific insertions in *Catarrhini* primates, further detailing the group’s dynamics while colonizing primate genomes. Moreover, hitherto unreported ERV elements closely related to ERV-W in *Platyrrhini* species provided important insights into putative ancestral sequence contributions.

## Results

### Comparative analysis of HERV-W orthologous loci in *Catarrhini* primates genome sequences

Subsequent to our recent characterization of 213 HERV-W loci in the human genome assembly hg19 [44], we now analyzed in detail the presence/absence of orthologous loci in the genome sequences of non-human primate species. For the sake of simplicity, we will refer to the respective non-human primate sequences as ERV-W, in order to distinguish them from the human (HERV-W) sequences. Making use of homologous genome regions and annotations provided by UCSC Genome Browser [47–49], the presence of HERV-W-orthologous ERV-W loci was examined in the genome sequences of Rhesus Macaque, Gibbon, Orangutan, Gorilla and Chimpanzee, by comparison of the respective ERV-W loci. To properly verify the presence of each ERV-W locus, we dedicated particular attention on nucleotide sequence similarity of the genomic regions flanking its insertion site. Of note, since no comparable sequence information was available for 2 HERV-W loci on chromosome Y, except for Chimpanzee, in our investigation we considered the remaining 211 HERV-W loci.

Our analysis generated an exhaustive comparative map of orthologous ERV-W insertions (Additional file 1: Table S1). Analysis of *Hominoidea* species Chimpanzee, Gorilla and Orangutan genome sequences revealed an overall number of orthologous ERV-W loci comparable to the one observed in human genome assembly GRCh37/hg19 [44], while Gibbon and Rhesus genome sequences harbored a lower number of orthologous ERV-W loci (Table 1). The absence of an entire ERV-W insertion in some primates could be due to an integration having occurred after the separation of the respective evolutionary lineages, thus providing direct information on the time period of germ line colonization. It could however also depend on deletions, rearrangements, errors in genome sequence assemblies or in their comparative analysis, particularly for primate species with less complete assemblies.

**Table 1:** Number of orthologous HERV-W loci in the analyzed Catarrhini primate genome sequences

	Chimpanzee	Gorilla	Orangutan	Gibbon	Rhesus
ERV-W loci orthologous to human 211^a^HERV-W elements	205	207	205	190	131

^a^no reliable sequence information was available for two HERV-W loci in human chromosome Y (see text)

**Fig. 2:**
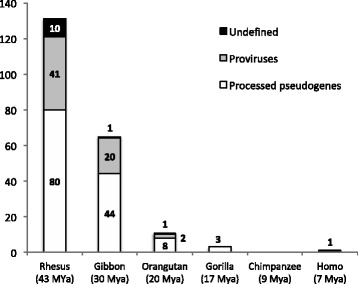
Initial formation of 211 HERV-W loci based on respective orthologs in *Catarrhini* primate reference genomes. The number of orthologs to HERV-W loci initially formed in a particular primate species is given for each species for proviruses, L1-retrotransposed processed pseudogene and undefined elements (see text for more details). For instance, 20 HERV-W loci were initially formed in the common ancestor of human and Gibbon, and 8 HERV-W processed pseudogenes were formed in the common ancestor of human and Orangutan. Note that the majority of HERV-W loci was initially formed in the common ancestor of human and Rhesus and is thus common to all *Catarrhini* genomes. Approximate time periods of last common ancestors of *Catarrhini* primate lineages are given in millions of years ago (MYa) below species names

Based on our analysis, 123 out of 211 (H)ERV-W loci are actually shared by all analyzed *Catarrhini* primates, from human to Rhesus. However, when considering also the (H)ERV-W loci found in Rhesus and human but apparently absent in some intermediate primates (see above), the number of shared ERV-W loci increases to 131/211 (Fig. 2). Those findings corroborate the view that the first and major wave of ERV-W loci formation occurred between 43 and 30 MYa, after the separation of *Catarrhini* and *Platyrrhini,* but before the divergence of Rhesus lineage from *Hominoidea*, in line with previously reported integration periods [44, 46, 50]. In addition to this first wave of formation, a total of 80 HERV-W loci was lacking an ortholog in Rhesus, but had orthologs only in subsequent *Hominoidea* species, suggesting the integration of about 66 novel HERV-W loci less than 30 MYa. Differently, relatively few insertions (14) likely occurred later on, between 20 and 17 MYa (Fig. 2).

Overall, (H)ERV-W insertions comparison in primate genome sequences indicated that the ERV-W group formed new loci throughout an extended period of time during evolution, due to both novel proviral integrations (*n* = 63) and L1-mediated processed pseudogene formations (*n* = 133). In particular, > 90% of ERV-W orthologs were acquired by Rhesus (*n* = 131) and Gibbon (*n* = 65), approximately between 43 and 20 MYa, showing in both species a 2:1 ratio of processed pseudogenes relative to proviruses. These data indicate that ERV-W processed pseudogene formation occurred during considerable extent of time, also implying that ERV-W transcripts serving as templates for L1 retrotransposition must have been present in the germ line during that period. A pronounced decline in ERV-W locus formation was then observed in Orangutan, with 8 and 2 novel ERV-W processed pseudogenes and proviruses, respectively; as well as in Gorilla, harboring 3 novel ERV-W processed pseudogenes and no new proviral integration. This suggests that L1-mediated formation of ERV-W loci occurred for an extended period of time when compared to true provirus formations, and also at significant extent in more recent primate lineages. Of further note, no new formations of ERV-W loci were observed in Chimpanzee, while a HERV-W locus in chromosome 12q13.3 appeared to be human-specific because of an empty site in the orthologous genome regions of all non-human *Catarrhini* primates, thus possibly suggesting that an HERV-W insertion has occurred less than 7 MYa [39, 40]. However, the human-specificity of this sequence is uncertain due to the overall highly mutated structure of the locus and the lack of LTRs, making sequence divergence-based age estimation very unreliable [44].

### Analysis of ERV-W sequences identified by sequence similarity searches in non-human *Catarrhini* identifies species-specific insertions

The above comparative analysis revealed an extended period of ERV-W loci formation throughout primates’ evolution, with evidently 80 novel insertions since the separation of Gibbon and human lineages. Thus, such an extended time period of ERV-W activity could likely have also resulted in species-specific insertions outside of the human evolutionary lineage, therefore lacking an orthologous locus in humans. To identify potential species-specific ERV-W insertions, we performed UCSC Genome Browser BLAT searches of *Catarrhini* primates genome sequences by using the assembled LTR17-HERV17-LTR17 RepBase HERV-W reference as a query. It is worth noting that this BLAT search approach identified a lower overall number of ERV-W loci in each non-human *Catarrhini* primate, suggesting that a proportion of ERV-W elements were not effectively detected (Table 2).

**Table 2:** Numbers and orthologs of ERV-W sequences identified by HERV17 BLAT searches in *Catarrhini* primate genome sequences

	Chimpanzee	Gorilla	Orangutan	Gibbon	Rhesus
1) ERV-W loci with HERV-W orthologs in human genome	138 (67%)	132 (64%)	122 (60%)	111(58%)	69 (53%)
2) ERV-W loci corresponding to human solitary LTRs (*n* = 19)	1 (17)	1 (17)	7 (10)	10* (8)	14* (0)
3) ERV-W loci present in human as non-canonical HERV-W (like)	29	27	24	21	20
4) ERV-W loci lacking an ortholog in human	3 (3)	5 (4)	8 (6)	4 (2)	68 (66)
TOTAL	171	165	160	145	168

1) Number of ERV-W elements with an orthologous locus among the 211 HERV-W loci: respective percentages are given in parenthesis. Two HERV-W loci on human chromosome Y were excluded from the analysis (see text)

2) Numbers of ERV-W elements corresponding to a solitary LTR at the orthologous human position. Numbers in parenthesis indicate the proviral insertions acquired in evolutionarily older primate species that were likewise a solitary LTR in the non-human primates analyzed. “*” indicates species with initial formations of proviruses that recombined to solitary LTRs in subsequent primate species: Gibbon (5) and Rhesus (14)

3) Numbers of ERV-W elements with an ortholog in the human reference genome sequence, yet being less similar to HERV-W. Those sequences were not identified as HERV-W elements in a previous analysis [68]

4) ERV-W loci absent in the orthologous human genome positions. Numbers in parenthesis indicate the proportion of species-specific insertions

We further investigated those different outcomes by comparing the orthologous ERV-W loci retrieved by both approaches with the additional ones retrieved by BLAT searches only. Results showed that only 53–67% of the ERV-W orthologs (Table 1) were effectively identified by BLAT searches (Table 2, first row). The remaining BLAT-identified ERV-W loci could be explained by three corresponding states in the human GRCh37/hg19 assembly: i) presence of a HERV-W solitary LTR (Table 2, row 2); ii) presence of HERV-W-like elements with somewhat lesser identity (~ 63% on average) to HERV17 (Table 2, row 3); iii) complete absence of HERV-W or HERV-W-like sequence (Table 2, row 4). Each of those three conditions was analyzed separately and results are described in the followings.

i. *ERV-W BLAT-identified sequences being solitary LTRs in the human genome.* In 19 instances, a solitary LTR annotated as LTR17 was found at the orthologous position in the human reference genome (Table 2, row 2, and Additional file 1: Table S2), suggesting a previous event of LTR-LTR homologous recombination that eliminated the internal portion and one LTR [51] from ERV-W proviral integrations that had occurred either in Rhesus (14) or Gibbon (5), in line with the group’s main period of germ line colonization. None of the solitary or corresponding proviral LTRs showed signatures of processed pseudogenes, that likely would have prohibited homologous recombination due to relatively short homologous sequences within remaining 5′ and 3′ LTR portions.
ii. *ERV-W BLAT-identified sequences corresponding to HERV-W-like elements with lesser identity to HERV17.* The here reported lower scoring HERV-W-like elements (Table 2, row 3; Additional file 1: Table S3) had not been identified as HERV-W loci by BLAT searches in our recent characterization of the group in the human genome [44]. A closer inspection of RepeatMasker annotations revealed that some of those loci were composed of stretches of other Gammaretrovirus-like HERVs (γHERVs) (such as LTR12F flanking HERV9, HERV30 and HERVIP10FH internal portions) in human genome sequence, while they were annotated as HERV17 in non-human primates. Also, some of these loci were previously identified as non-canonical HERV9 elements, which are in fact closely related to the HERV-W group [38].
  Interestingly, ~ two-thirds of the HERV-W-like loci are present at orthologous positions ranging from Rhesus to human, having thus been likely formed during the main period of the (H)ERV-W group’s activity. The remaining (H)ERV-W-like elements presumably entered primate genomes only in the evolutionarily separated lineages leading to Gibbon (3), Orangutan (2), and Gorilla (2), while no novel elements were observed for Chimpanzee, as already observed for HERV-W orthologous loci.
iii. *ERV-W BLAT-identified sequences lacking an ortholog in humans*. A number of ERV-W loci identified by BLAT searches in non-human *Catarrhini* species lacked orthologous loci in the human genome (Table 2, row 4 and Additional file 1: Table S4). In theory, such ERV-W loci may have formed species- or lineage-specifically, and thus they could also provide information on the ERV-W group’s time period(s) of activity (Fig. 1). Interestingly, the great majority (81/88) of these ERV-W sequences are actually species-specific insertions (Additional file 1: Table S4), also suggesting an extended period of ERV-W germ line colonization in primates. In particular, 77% of ERV-W insertions in Rhesus appeared to be absent in humans, with still 66/68 species-specific elements when compared to non-human primate species more closely related. This further indicates that the main period of ERV-W activity ranges from 43 MYa to < 20 MYa, with a greater number of Rhesus-specific ERV-W acquisitions after the separation of its evolutionary lineage. The other non-human *Catarrhini* primates likewise showed some evidence for ERV-W insertions lacking a human ortholog: 4 loci in Gibbon (2 species-specific); 8 loci in Orangutan (6 species-specific); 5 loci in Gorilla (4 species-specific) and 3 in Chimp, (all species-specific) (Table 2, row 4 and Additional file 1: Table S4).
  Also noteworthy, Rhesus and Gorilla showed 15 and 1 new proviruses, respectively, suggesting that the ERV-W species-specific colonization has in part been due to either intracellular provirus formations or re-infections, likely hinting at sporadic acquisition of novel elements during the recent 10–5 MY. Similarly, species-specific formations of ERV-W processed pseudogenes in Rhesus (24), Orangutan (3), Gorilla (1) and Chimpanzee (1) further suggest that L1 retrotransposition of ERV-W transcripts has also been ongoing for considerable time periods outside of the human lineage, approximately between 43 and 5 MYa.

### Sequences closely related to HERV-W in *Platyrrhini* (new world monkeys)

The UCSC Genome Browser BLAT search in *Platyrrhini* species Marmoset (*Callithrix jacchus*) and Squirrel Monkey (*Saimiri boliviensis*) did not identify true ERV-W insertions, confirming that the group spread has been limited to *Catarrhini*. However, our searches identified a group of apparently highly related sequences, indicated as ERV1–1_CJa-I and ERV1–1_CJa-LTR for the internal portion and the 5′ and 3′ LTRs, respectively, based on RepBase annotations. For sake of brevity, those sequences will be referred to as ERV1–1.

**Fig. 3:**
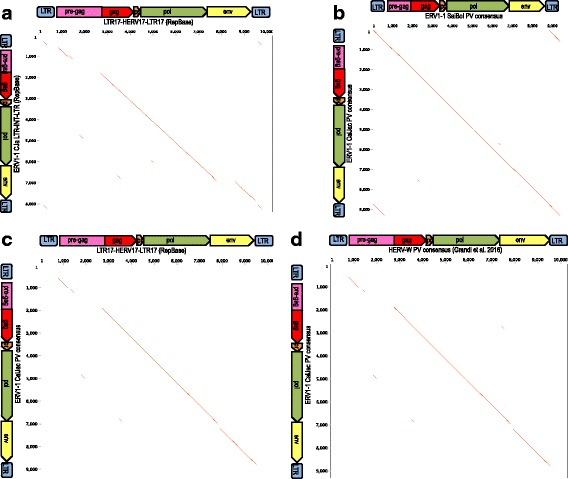
Pairwise nucleotide sequence comparisons depicting sequence similarities between HERV-W and ERV1–1 groups. Reference sequences and consensus sequences were compared with each other as follows. **a** *Callithrix jaccus* ERV1–1 RepBase sequence and HERV-W RepBase sequence; **b** *Callithrix jaccus* and *Saimiri boliviensis* ERV1–1 proviral consensus sequences as generated in this paper; **c** *Callithrix jaccus* ERV1–1 proviral consensus as generated in this paper and HERV-W RepBase reference sequence; **d** *Callithrix jaccus* ERV1–1 proviral consensus sequence as generated in this paper and a HERV-W proviral consensus as reported recently [44]. Sequence similarities in dot-plot comparisons are highlighted for sequence regions with at least 50% similarity along a 100 nucleotides sequence window. Proviral gene and LTR regions are depicted

Sequence similarities of HERV-W and ERV1–1 were further examined at the nucleotide level by the comparison of representative proviral sequences (Fig. 3). The pairwise comparison between the ERV1–1 and HERV-W RepBase references, assembled as LTR-internal-LTR, revealed an overall 73% sequence identity between internal portions (~nt 2700 to 7750 in the HERV-W sequence), albeit a portion of the HERV-W *env* gene (~nt 7750 to 8570) appeared to be absent in the ERV 1–1 reference (Fig. 3a). We further investigated ERV1–1 sequences by retrieving reasonably complete ERV1–1 proviruses, based on chromosome coordinates obtained from BLAT searches plus 5 kb of upstream and downstream flanking sequence each. The collected ERV1–1 sequences were analyzed for the presence of 5′ and 3′ LTRs, and the actual complete ERV1–1 proviruses from Marmoset (59) and Squirrel Monkey (71) assemblies were used to generate two species-specific multiple alignments and, subsequently, two majority rule-based consensus sequences, named ERV1–1_CalJac_PVconsensus and ERV1–1_SaiBol_PVconsensus, respectively (Additional file 2). Those consensus sequences were subjected to dot-plot comparison and pairwise alignment to assess differences between the ERV1–1 groups in the two NWM species (Fig. 3b). Since the two consensus sequences showed 98% overall identity, the ERV1–1_CalJac proviral consensus was chosen as representative for both species for subsequent analysis. Comparison of ERV1–1_CalJac proviral consensus with the HERV-W RepBase reference (Fig. 3c) and the HERV-W consensus previously built from the human proviral dataset [44] (Fig. 3d) revealed that the above mentioned *env* portion was not represented in the ERV1–1 RepBase reference due to a larger deletion within the concerned *env* gene region in the majority of ERV1–1 sequences, similar to a recurrent structural variant in approximately 80% of HERV-W elements [44]. Inclusion of this often-missing *env* portion in the ERV1–1_CalJac proviral consensus sequence thus confirmed the high sequence identity with HERV-W along the full-length *env* gene. Interestingly, the comparisons showed that ERV1–1 sequences also harbor a so-called “*pre-gag”* region between the 5′ LTR and the *gag* gene, as previously reported for HERV-W elements (~nt 800 to 2700 in LTR17-HERV17-LTR17) [44]. Of further note, contrary to the proviral internal portion, ERV1–1 LTRs did not show pronounced similarity (overall 34%) to either the LTR17 RepBase sequence or the proviral HERV-W LTR consensus. Accordingly, BLAT searches did not identify sequences resembling LTR17 in Marmoset or Squirrel Monkey genomes.

### Presence of ERV-W related elements in other NWM families

To the best of our knowledge, unlike Marmoset and Squirrel Monkey, no genome sequence assemblies are available for the other two *Platyrrhini* families, *Atelidae* and *Pitheciidae*. We therefore performed BLAST searches of unassembled sequences of Spider Monkey (*Ateles geoffroyi*, *Atelidae* family) and Red-bellied Titi (*Callicebus moloch*, *Pitheciidae* family) available in the NCBI Trace Archive database, using both LTR17-HERV17-LTR17 and ERV1–1_CalJac proviral consensus sequence as queries. Results confirmed the presence of ERV1–1 elements highly related to ERV-W internal portion also in these two NWM families (data not shown).

### Absence of elements closely related to ERV-W in Tarsiiformes and Prosimians

To complete our search for ERV-W-related sequences, we performed BLAT searches in UCSC Genome Browser assemblies of species representative for *Tarsiiformes*, i.e. Tarsier (*Tarsius syrichta*), and *Prosimians*, i.e. Bushbaby (*Otolemur garnettii*) and Mouse Lemur (*Microcebus murinus*). Only short matches with insignificant scores were retrieved, indicating the absence of ERV-W-related elements in those species (data not shown) and further confirming that their spread took place after the evolutionary separation of *Prosimians* and *Simiiformes*, occurred ~ 60 MYa [39, 40].

### Analysis of retroviral puteins corroborate close relationship of ERV1–1 with the ERV-W group

**Fig. 4:**
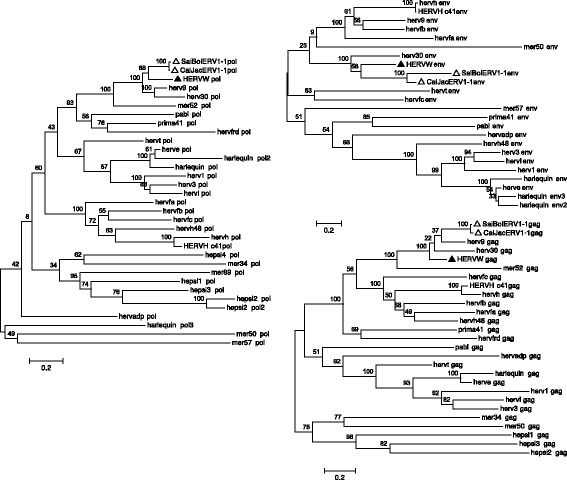
Phylogenetic analysis of ERV1–1 Gag, Pol and Env puteins. ERV1–1 puteins, labeled with an empty triangle, were obtained by identification and conceptual translation of Marmoset ERV1–1 proviral consensus sequence Open Reading Frames (see methods). The other Gammaretroviral putein sequences were retrieved from Vargiu et al. 2016 [38]. HERV-W puteins are marked with a filled triangle. The evolutionary relationships were inferred by using the ML method based on the Poisson model. Phylogenies were tested by using the bootstrap method with 100 replicates each: the obtained bootstrap values are reported near each node (bootstrap values lower than 30% are not shown). Length of branches indicates the number of substitutions per site

To further characterize sequence relationships between ERV1–1 and ERV-W groups, we analyzed their phylogeny with respect to other endogenous and exogenous Gammaretroviruses [38, 52] at the amino acid level, by using Maximum Likelihood (ML) analysis of Gag, Pol and Env putative proteins (puteins) (Fig. 4). To this aim, ERV1–1 ORFs were identified in Marmoset and Squirrel Monkey ERV1–1 proviral consensus sequences by the software RetroTector [37], reconstructing the amino acid sequences of encoded retroviral puteins. Subsequent ML analysis revealed that both ERV1–1 Pol and Env puteins were most closely related to the HERV-W puteins, further demonstrating a strong evolutionary relationship between those groups. A less pronounced relationship was found for the Gag putein (Fig. 4), even if ERV1–1 Gag sequence was one of the best hit identified by RetroTector for HERV-W Gag recognition [38]. It is interesting to note that, even if HERV-W appears to be a closer relative to ERV1–1, ERV1–1 puteins clustered also with other Gammaretrovirus-like families known to be related to HERV-W, such as HERV9 and HERV30, possibly further hinting towards a common evolutionary origin of all those (H)ERV groups.

### Phylogeny and ERV1–1 sequence relationships with human solitary LTRs and HERV-W-like elements and with *Catarrhini* ERV-W elements without human orthologs

To further characterize the elements identified by BLAT searches in the *Catarrhini* non-human primate genomes and lacking orthologs in humans, the above mentioned three subsets of sequences were compared with the consensus sequences generated for HERV-W [44] and ERV1–1 and the reference sequences of other γHERVs as provided by RepBase.

**Fig. 5:**
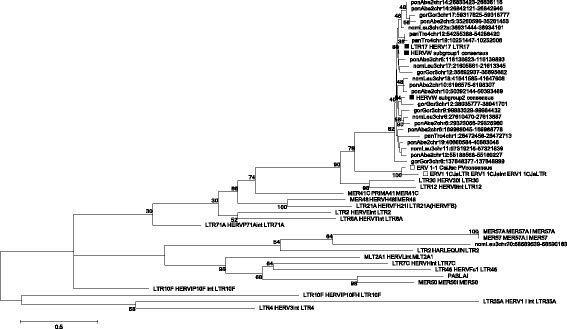
Phylogenetic analysis of Chimpanzee, Gorilla, Orangutan and Gibbon ERV-W nucleotide sequences lacking an ortholog in the human genome. Gammaretrovirus-like HERV reference sequences were retrieved from RepBase. For the HERV-W group, both RepBase reference and the consensus sequences generated previously from the proviral dataset [44] were included and marked with a filled square. The ERV1–1 reference sequence from RepBase and the consensus generated from the proviral sequences dataset in this study are marked with an empty square. Evolutionary relationships were inferred by using the ML method and the Kimura-2-parameter model. The resulting phylogeny was tested using the bootstrap method with 100 replicates: the obtained bootstrap values are reported near each node (bootstrap values lower than 30% are not shown). Length of branches indicates the number of substitutions per site

i) *ERV-W BLAT-identified sequences being solitary LTRs in human.* ML phylogenetic analysis of human solitary LTRs derived from ERV-W proviral insertions in Rhesus (14) and Gibbon (5) confirmed that they belong to the HERV-W group, clustering with the LTR17 consensus (100% bootstrap support) and being clearly separated from all other γHERV sequences (Additional file 3).
ii) *ERV-W BLAT-identified sequences corresponding to HERV-W-like elements with lesser identity to HERV17.* ML phylogenetic analysis of HERV-W-like elements with lower nucleotide identity to HERV17 revealed three clusters of sequences with reasonable bootstrap support: cluster I, 96%; cluster II, 100%; cluster III, 70% (Additional file 4). These three clusters were separated from the other γHERVs with a 96% bootstrap support and included 24 out of 29 HERV-W-like sequences as well as HERV-W, HERV9, HERV30 and ERV1–1 references. Cluster I elements were most related to HERV-W, while cluster II sequences showed closer relationships to HERV9 and HERV30 (Additional file 4). In accord, RepeatMasker analysis (Additional file 1: Table S3) confirmed that cluster I members were annotated exclusively as HERV17. Cluster II members included elements structurally related to HERV17 and, in one case, HERV30 in the internal portions, yet harboring LTR12F (the HERV9 LTR in RepBase) as LTR type. Cluster III members were indeed only remotely related to the other HERV-W-like elements (bootstrap support = 52), being clearly separated from γHERVs (Additional file 4). RepeatMasker analysis, however, identified these sequences either as LTR17 and HERV17 or as other related γHERVs (HERV9, HERV30, HERVH, HERVIP10FH) (Additional file 1: Table S3). Overall, these results demonstrated closer relationships, yet of different degrees, of HERV-W-like elements with HERV-W, HERV9, HERV30 and ERV1–1.
iii) *ERV-W BLAT-identified sequences lacking an ortholog in human*. To verify the phylogeny of *Catarrhini* ERV-W sequences lacking an ortholog in humans with respect to the other γHERV sequences, Chimp, Gorilla, Orangutan and Gibbon full-length sequences were analyzed separately (Fig. 5) from Rhesus ERV-W sequences, whose phylogeny was inferred considering the *pol* gene only because of the relatively high number of elements (Additional file 5).

All ERV-W sequences identified in Chimpanzee, Gorilla, Orangutan and Gibbon grouped with the HERV-W consensus (82% bootstrap) and were furthermore closely related to ERV1–1 (78% bootstrap) followed by HERV9 and HERV30 (Fig. 5). A single sequence retrieved from Gibbon (chr20:58,589,539–58,590,163) displayed a rather weakly supported (64%) relationship with MER57.

The separately analyzed Rhesus ERV-W *pol* sequences likewise formed a well-supported (90%) cluster with HERV-W (Additional file 5). That phylogenetic clade was likewise related to HERV9 and HERV30 with high bootstrap supports (99%). Six Rhesus ERV-W sequences were instead located outside of that cluster. Those sequences’ actual nature was further examined by comparing their full-length nucleotide sequences to a subset of γHERV reference sequences by EMBOSS polydot analysis (Additional file 6). Particularly, a sequence related to MER57 in ML tree (chr4:4,004,556–4,011,519; 64% bootstrap) shared longer stretches of identity exclusively with the HERV-W consensus sequence. Four other sequences that clustered together with 100% bootstrap support and were furthermore weakly related to HERV-H (31% bootstrap) displayed longer stretches of similarity with both HERV-W and HERV-H consensus sequences, possibly representing non-canonical mosaic forms. Another sequence forming a separate branch in ML tree (chr1:51,551,811–51,557,699) did not show appreciable similarity to any of the γHERV sequences (Additional file 6).

Taken together, phylogenetic analysis confirmed the ERV-W nature of almost all the retrieved ERV-W-like elements without human orthologs in non-human *Catarrhini* species as well as the independent spread of “true” (H)ERV-W elements in Rhesus later in primate evolution.

## Discussion

Following up on our recent characterization of the HERV-W group in the human genome [44], the present work aimed to analyze the ERV-W elements integrated in genome sequences of non-human primates, to provide a complete and definitive depiction of the group spread during primates evolution. A number of studies, in fact, suggested that the initial ERV-W colonization of primate’s germ line had occurred in *Catarrhini* after their evolutionary separation from *Platyrrhini*, i.e. < 40 MYa, based on results from HERV-W *pol* PCR [53] and Southern Blot [50] analysis of different non-human primates samples, or from the nucleotide divergence between HERV-W subfamilies [46]. Such results were supported by the absence of ERV-W sequences in *Platyrrhini* and *Prosimians* [46, 50, 53]. One of these works reported, in addition, the presence of solitary ERV-W LTRs also in three *Platyrrhini* species based on PCR results, suggesting that ERV-W LTR acquisition occurred approximately 55 MYa [53]. Overall, the previously available information suggests that the first (H)ERV-W proviral acquisitions occurred around 25 MYa, and the group as a whole formed during a rather short period of activity (~ 5 MY) [46, 50, 54]. Such relatively low proliferation rate had been explained by the abundance of HERV-W L1-processed pseudogenes, being proliferation-incompetent due to the lack of 5’LTR U3 and 3’LTR U5 regions [46].

Our detailed analysis of primate genome sequences provided the definitive support that the ERV-W group is present exclusively in *Catarrhini* primates. However, our searches for ERV-W orthologous loci in the genomes of Hominoids and OWMs revealed that the group proliferated for an extended time period, with novel locus formations having occurred approximately between 43 and 20 MYa, in line with recent age estimates of single HERV-W sequences [44]. Interestingly, a 2:1 ratio of L1-mediated processed pseudogene formations relative to “true” provirus formations was observed in Rhesus and Gibbon, suggesting that a quite massive formation of ERV-W processed pseudogenes likewise occurred during an extended time period. Similarly, ERV-W processed pseudogenes were the main source of additional ERV-W locus acquisitions also in Orangutan and Gorilla.

The spread of the ERV-W group within the parvorder *Catarrhini* was further investigated through BLAT searches at the UCSC Genome Browser, using the RepBase HERV17 reference sequence as a query. That strategy identified 4 ERV-W loci in Gibbon and 15 in Rhesus that were likely formed between 43 and 20 MYa and were present in the human genome only as solitary LTRs. BLAT searches furthermore identified 29 ERV-W-like elements with somewhat lower similarities to HERV-W, mostly present in the Rhesus genome but also found in Gibbon (3), Orangutan (2) and Gorilla (2).

In support of a longer time period of ERV-W locus formations, some ERV-W loci in non-human primates appeared to be species-specific and thus lack orthologs in the other species. In particular, we identified 88 ERV-W loci with corresponding empty sites in the human genome, 81 of which could be interpreted as species-specific insertions in respective primates: 66 in Rhesus, 2 in Gibbon, 6 in Orangutan, 4 in Gorilla, and 3 in Chimpanzee. The latter further indicate lineage-specific formations of ERV-W loci less than 10 MYa. Importantly, species-specific acquisition of ERV-W loci occurred by both full-length proviruses and L1-mediated processed pseudogenes formation. It should be stressed here that our analysis of (orthologous) ERV-W loci present (or absent) in the various available primate genome sequences relies on comparative genomics data as provided by the UCSC Genome Browser [49, 55] and required a minimum of 500 nt of upstream and downstream flanking sequences to ensure analysis of truly homologous genome regions. While some of the observed differences in orthologous ERV-W loci may be due to errors in genome sequence assemblies or (b)lastz alignments, it appears that only a minority of loci are associated with, or in close proximity to, for instance, gaps in assembled genome sequences.

Taken together, our comparative analysis of primate genome sequences thus provides a detailed evolutionary history of (H)ERV-W sequences and their spread during *Catarrhini* evolution, corroborating an extended period of ERV-W locus formations, having peaked between ~ 42 and 30 MYa, and providing sporadic, species or lineage-specific ERV-W locus formations until < 10 MYa, confirming the absence of ERV-W sequences in NWMs regarding neither gene regions nor LTRs.

Of note, our sequence searches identified an ERV group closely related to ERV-W, named ERV1–1_CJa in RepBase. Because of the lack of an established ERV nomenclature, we designated those sequences as ERV1–1. A total of 130 ERV1–1 loci were identified in the genomes of Marmoset (59) and Squirrel Monkey (71), and searches of unassembled genome sequence data furthermore indicated the presence of ERV1–1 sequences in species belonging to all the three *Platyrrhini* families. However, there was no evidence of ERV1–1 sequences in *Tarsiiformes* and *Prosimians*, indicating that their formation in the respective primate lineage occurred < 60 MYa based on estimated times of separations of respective lineages [39, 40]. Also noteworthy, despite the remarkable identity along the proviral internal portion, none of the ERV1–1 loci showed signatures of processed pseudogenes, as it is the case for many (H)ERV-W loci [45, 46], suggesting a central role of LTRs in L1-recognition and retrotransposition of (H)ERV-W transcripts. The established close sequence relationships at both nucleotide and amino acid level suggest that ERV1–1 and (H)ERV-W could derive from a common ancestor, possibly also involving related groups such as HERV9 and HERV30. As mentioned above, such closer sequence relationships do not apply to the ERV1–1 LTRs, that appear very different in sequence from (H)ERV-W LTRs. This is however in line with previous observations in other ERV groups for which different paths of evolution were taken by the proviral body and the LTR sequences, resulting in different LTR subgroups associated with otherwise monophyletic proviral bodies (for instance, see [56, 57]) and possibly leading to retroviral chimeras formation [38].

Given the relatively recent availability of many eukaryotic genome sequences and new bioinformatics tools, the field of paleovirology is currently emerging. In this view, ERVs may have a central role in understanding the evolution of both host and virus. Regarding host evolution, as described in the introduction, ERVs significantly contributed to the host genome shaping by introducing genetic variation and novel functions. In addition, as it has been shown in the case of retroviruses with an ongoing process of endogenization, such as the Koala retrovirus (KoRV) [34], there is a complex dynamics of retroviral/host evolution suggesting that ERV acquisition may be an effective defence strategy against exogenous viral pathogenic infections [58]. Hence, the present study set the basis for further analysis of the role of specific ERV-W sequences in primates, providing for the first time exhaustive information regarding both the individual loci shared by different species and the ones acquired exclusively by one of them. Regarding viral evolution, our results showed unprecedented similarities between ERV-W and ERV1–1 sequences, providing unreported insights on their evolution and describing in greater detail the dynamics of the ERV-W group’s spread regarding ancient orthologous insertions that are shared by primates including human, as well as species-specific ERV-W locus formed in non-human primates. Those findings, combined with a reasonably accurate estimation of the times of integration through a combined approach, now provides a complete overview of the ERV-W group’s colonization of primate genomes and may allow to better understand the complex history of acquisition, cross-species transmission and clade-specific amplification that have been shaped by host, viral, and ecological factors [59].

Our study leaves also room for some speculations that deserve further investigation. For example, the fact that the majority of ERV-W sequences are shared by all the analysed primates might suggest a relevant role of the ERV-W group in the ancestral infected population, that could possibly has been favoured in bottleneck events by the protection against deleterious exogenous infections, as seen for KoRV, or some other advantages. Similarly, the species-specific insertions could instead have provided, at least temporarily, specific advantages for those species and lineages.

It is also worth mentioning that ERV-W locus acquisitions in primates by L1-mediated processed pseudogene formation during an extended period of time provided novel insights into the mechanisms of the ERV-W group’s copy number increases and proliferation activity, further highlighting the special link between ERV-W and L1 [60, 61]. The latter is still poorly understood, especially regarding the specific molecular determinants that limited the L1-retroposition to (H)ERV-W transcripts only, without involving any other (H)ERV groups [43].

## Conclusions

The present study offers an exhaustive overview of the germ line colonization of ERV-W during the evolution of primates, revealing a rather unexpectedly long period of activity and several species-specific activation and providing novel insights on the evolution of the group and its close unreported relation with NWMs ERV1–1 elements. It also characterized the contribution of other human TEs to the spread of ERV-W in primates, pointing out that L1-mediated formation of ERV-W processed pseudogenes was not a secondary phenomenon with negative impact on the group’s proliferation rate, but instead a parallel and major mechanism of ERV-W locus formations in all primates genomes.

## Methods

### Sequence collection

*1) HERV-W orthologous ERV-W sequences in non-human Catarrhini primate genome sequences.*

Identification and collection of ERV-W sequences orthologous to previously characterized HERV-W loci was done by using information provided by the UCSC Genome Browser [49, 55] for the following non-human *Catarrhini* primate genome sequence assemblies:

- Chimpanzee (*Pan troglodytes*, assembly Feb. 2011 - CSAC 2.1.4/panTro4)
- Gorilla (*Gorilla gorilla gorilla*, assembly May 2011 - gorGor3.1/gorGor3)
- Orangutan (*Pongo pygmaeus abelii*, assembly July 2007 - WUGSC 2.0.2/ponAbe2)
- Gibbon (*Nomascus Leucogenys*, assembly Oct. 2012 - GGSC Nleu3.0/nomLeu3)
- Rhesus (*Macaca mulatta*, assembly Oct. 2010 - BGI CR_1.0/rheMac3)

Comparative analysis of presence or absence of HERV-W orthologous loci involved examination of a minimum of 500 nt of 5′ and 3′ flanking genomic sequence in respective primate genome sequences.

*2) ERV-W sequences in non-human Catarrhini primate genome sequences.*

Additional ERV-W sequences in non-human *Catarrhini* primate genomes sequence assemblies were identified by BLAT searches [62] at the UCSC Genome Browser [49, 55] using an assembled sequence consisting of LTR17-HERV17-LTR17 as provided by RepBase [63] as a query. The so identified ERV-W loci were mapped to the human genome to investigate the presence of orthologous elements, by using UCSC Genome Browser comparative genomics, as described above. Absence of a HERV-W sequence in an orthologous genome region was concluded when no HERV-W sequences were found by BLAT searches using HERV17 and the ERV-W nucleotide sequence from the respective orthologous primate genome region (including flanking genomic regions) as queries.

*3) ERV-W-related ERV1–1 sequences in Platyrrhini primate genome sequences.*

ERV-W-related ERV1–1 elements were identified by a UCSC Genome Browser BLAT search, using the RepBase HERV17 sequence as a query, in the following *Platyrrhini* primates (family *Cebidae*):

- Marmoset (*Callithrix jaccus*, assembly March 2009 - WUGSC 3.2/calJac3)
- Squirrel Monkey (*Saimiri boliviensis*, assembly Oct. 2011 - Broad/saiBol1)

ERV1–1 sequences were retrieved including 500 nucleotides 5′ and 3′ flankings, and proviruses with relatively intact LTRs based on pairwise dot-plot comparison were selected for subsequent analysis.

Since no assembled genomes sequences were available for representative members of the other two *Platyrrhini* families, i.e. *Atelidae* and *Pitheciidae*, the presence of ERV-W-related elements was assessed by BLAST searches of unassembled genomic sequence data available from the NCBI Trace Archive database (https://trace.ncbi.nlm.nih.gov/Traces/sra/sra.cgi?) for:

- Spider Monkey (*Ateles geoffroyi*, *Atelidae* family),
- Red-bellied Titi (*Callicebus moloch*, *Pitheciidae* family)

using LTR17-HERV17-LTR17 and a majority-rule ERV1–1 consensus (Additional file 2) as queries.

*4) ERV-W-like sequences in Tarsiiformes and Prosimian genome sequences.*

ERV-W-like elements were searched by UCSC Genome Browser BLAT using LTR17-HERV17-LTR17 and a majority-rule ERV1–1 consensus (Additional file 2) as queries in the following species:

- Tarsier (*Tarsius syrichta*, *Tarsiiformes*, assembly Sep. 2013 – Tarsius_syrichta-2.0.1/tarSyr2)
- Bushbaby (*Otolemur garnettii, Lemuriformes*, assembly Mar. 2011 – Broad/otoGar3)
- Mouse Lemur (*Microcebus murinus, Lorisiformes*, assembly Jul. 2007 – Broad/micMur1)

### Pairwise and multiple alignments of sequences

Multiple alignments of nucleotide and amino acid sequences were generated by Geneious software, version 8.1.4 [64] using MAFFT algorithms FFT-NS-i × 1000 or G-INS-I [65] with default parameters. All multiple alignments were visually inspected and, when necessary, manually optimized before subsequent analysis. Sequences pairwise comparisons were done using the Geneious dot-plot tool Graphical depictions of alignments were generated with Geneious and further adapted manually.

### Phylogenetic analysis

*1) Phylogenetic trees.*

All phylogenetic trees were built from manually optimized multiple alignments (see above) by MEGA software, version 6 [66] using Maximum Likelihood (ML) or Neighbor Joining (NJ) methods. For nucleotide alignments: ML trees were built using the Kimura 2-parameter model, and phylogenies were tested by the bootstrap method with 100 replicates. For amino acid alignments: ML trees were built using the Poisson correction model, and phylogenies were tested by the bootstrap method with 100 replicates; while NJ trees were built using the Poisson correction model after applying pairwise deletion of missing sites, and phylogenies were tested by the bootstrap method with 1000 replicates.

See figure legends and the manuscript text for further details on specific phylogenetic analysis.

2) *Calculation of pairwise nucleotide distances.*

Pairwise divergence between aligned nucleotide sequences was estimated by MEGA Software, version 6 [66] using p-distance model and pairwise deletion after removal of CpG dinucleotides,

### ERV1–1 ORFs and prediction of putative proteins (puteins)

ERV1–1 Gag, Pol and Env amino acid sequences were obtained from the bioinformatics reconstructions of retroviral ORFs and puteins in a majority-rule ERV1–1 consensus (Additional file 2), by using i) ReTe online version (http://retrotector.neuro.uu.se/pub/queue.php?show=submit) [67], ii) Geneious software [64] ORF finder and three-frame translations functions.

## Additional files

Additional file 1: Table S1. HERV-W loci in the human reference genome sequence and ERV-W orthologous sequences in non-human *Catarrhini* primates reference genome sequences. **Table S2**: ERV-W loci in non-human *Catarrhini* primate reference genome sequences with a solitary HERV-W LTR at the orthologous human genome position. **Table S3**: ERV-W loci in non-human *Catarrhini* primates corresponding to HERV-W-like elements with lesser similarities to HERV17. **Table S4**: ERV-W loci in non-human *Catarrhini* primate genome sequences lacking an ortholog in the human reference genome sequence. (XLSX 85 kb)

Additional file 2: *ERV1–1 consensus sequences in FASTA format. (DOCX 187 kb)*

Additional file 3: *Phylogenetic analysis of human solitary LTRs orthologous to ERV-W loci formed in Rhesus or Gibbon.* Gammaretrovirus-like HERV LTR sequences were retrieved from RepBase: the HERV-W group LTR17 reference sequence is marked with a filled square. The ERV1–1 LTR consensus were generated from the Marmoset (CalJac) and Squirrel Monkey (SaiBol) proviral sequence datasets, and are marked with empty squares. Evolutionary relationships were inferred by using the ML method and the Kimura-2-parameter model. The resulting phylogeny was tested using the bootstrap method with 100 replicates: the obtained bootstrap values are reported near each node (bootstrap values lower than 30% are not shown). Length of branches indicates the number of substitutions per site. (PDF 15 kb)

Additional file 4: *Phylogenetic analysis of HERV-W-like nucleotide sequences orthologous to ERV-W loci identified in non-human primates by HERV17 BLAT searches.* Gammaretrovirus-like HERV reference sequences were retrieved from RepBase. The HERV-W group RepBase LTR17 HERV17 LTR17 reference sequence and the proviral HERV-W subgroup 1 and 2 consensus sequences generated previously [44] are marked with a filled square. The ERV1–1 reference sequence from RepBase and the consensus generated from the proviral sequence dataset in this study are marked with an empty square. Evolutionary relationships were inferred by using the ML method and the Kimura-2-parameter model. The resulting phylogeny was tested using the bootstrap method with 100 replicates: bootstrap values are reported near each node (bootstrap values lower than 30% are not shown). Length of branches indicates the number of substitutions per site. (PDF 20 kb)

Additional file 5: *Phylogenetic analysis of pol gene nucleotide sequence from Rhesus ERV-W loci lacking an ortholog in the human reference genome.* Gammaretrovirus-like HERV *pol* gene reference sequences were retrieved from RepBase. The HERV-W group *pol* sequences from RepBase reference sequence and the proviral HERV-W consensus sequence generated previously [44] are marked with a filled square. The ERV1–1 *pol* sequences from RepBase reference sequence and the consensus generated from the ERV1–1 sequences dataset in this study are marked with an empty square. Evolutionary relationships were inferred by using the ML method and the Kimura-2-parameter model. The resulting phylogeny was tested using the bootstrap method with 100 replicates: bootstrap values are reported near each node (bootstrap values lower than 30% are not shown). Length of branches indicates the number of substitutions per site. (PDF 23 kb)

Additional file 6: *Polydot pairwise analyses of the 6 Rhesus ERV-W nucleotide sequences lacking an ortholog in the human reference genome sequence and showing unclear sequence relationships with other HERV sequences.* Analyzed consensus sequences marked “*” were generated in this study. Other sequences were retrieved from RepBase. (PDF 174 kb)

## Abbreviations

Env: Envelope
ERVS: Endogenous retroviruses
HERVs: Human endogenous retroviruses
L1: Long interspersed nuclear elements 1
LTRs: Long terminal repeats
ML: Maximum likelihood
Mya: Millions of years ago
NWM: New world monkeys
ORFs: Open reading frames
OWM: Old world monkeys
TEs: Transposable elements
γHERVs: Gammaretrovirus-like HERVs
