# Supplementary material for: HERV-W group evolutionary history in non-human primates: characterization of ERV-W orthologs in Catarrhini and related ERV groups in Platyrrhini
Source: BMC Evol Biol. 2018 Jan 19;18:6. doi: 10.1186/s12862-018-1125-1 (PMC5775608; doi:10.1186/s12862-018-1125-1)
Supplement: Supplementary file 2 — ERV1–1 consensus sequences in FASTA format. (DOCX 187 kb) [file 12862_2018_1125_MOESM2_ESM.docx]

ERV1-1 FASTA consensus sequences generated from our dataset of complete ERV1-1 proviruses retrieved from Marmoset (CalJac) and Squirrel Monkey (SaiBol) genome sequences, respectively.

For each species, the full-length proviral consensus sequences (_PVconsensus) are provided as assembled LTR-INTERNAL-LTR portions. The separate FASTA consensus for LTR and internal portions are also reported (PVconsensus_LTR and PVconsensus_INTERNAL, respectively).

>ERV1-1_CalJac_PVconsensus

TGAGGGACTGTGTCTCTTCCCGGGTCCACAAGGGGCGTGGTTTCAAAGGCAAGAATTTCCCAGCTCCATGCCTCCATCCAGGTCCCAGAGGGGTTTCAAATGTAAAGTTCTTTGTCTAACTTTCCCGCGCGCGGGCTAGGTAGAGAAATCCAGGTGAGCTTCCTTTGTTCAAAAAGCCCACCAAAAGCTCTGGGAGGAGGGGGCTCTGTTGTGGACCAAGCAGCCAATCCAGTATCAGAGTCAAAGATCAATCACTCCAGAGGAAGTTTGAAAGAACTCCTCTCATTGGACGAGAACATGAATGGGGAGGGAATAACTCCAGGGTTAAAATTCCACTGCCCCTTACCTATATGGATTTCCCCTCTGGGWTCCCCTCCCACAACAGCGTGGGAGCTGTCTCTCTCTCTCTCTCTCTCTCTCTCTCTGCCTAATAAATTCCTTTTCTGCAAAACTCTTTGGGTCCTGATATTTATTTGAACGGTAAGCTCACCGCACCTCCAGGGCCCCTTTCCCCATTCTTGGGGGTAAGAGAGGCCAAGAACCTGGAAAATCTGGGAACTGAGAGCATCCTCCTCCAGTTACATCTTTGGCGAGCCAGCCAGGAGACATCAGACTTCAGAGTGGTGAGTGATATTGGACCCCTTTCACTTGCTAATCTGTCCTTTCCCTTCTTTTAAATTTGTTAAGGAGGTAAAGACTGGGCACCTGTCAGCCATCTAAGAGCGACTAGCGTAGCCACCAGGCTAAAGACACGGGTGTCAGGCTTTCTGGGAACGGGCTCTCTAGCAACCCCCAGCTTTTCAGAACTGGGAGCATTGGTTAGCCCTGAACCAGCTTCCCTTTCTTGCTGCTCTTTCTTTCTCTGGGCCAAGCCGAGGGCCGGCAGGAGGAAAGGCGGTCCCAAACTCCCTGTTCAGTCCAGTTGAGGTTACGGCCCTGCTTAGACCTCCTAAGAAAAAGCCGCCTATGCGAGCCATGCCCAGCCTTTCAGACCCCAACCTCCTGGGTCATTTCCATGAGGCAAACTTCTTCCCGGCTCTTTCCTCTAAGATATTTCCTACACATGAGGCTATTACCCTTCTTGCTAGGACTTTGAGGCTCTTGGGGTGGCAGTCCAGGGACCCTTGCCCATGGTGCCCTCAGGCTTATGCCTATGAGGACCTCCTACAGTATAAATGCACAGGGCCAGTTCCCTTAGTCTGCAGTGCTAGGTTATGTCCTAACAGAGCCAGAGTCTATCAGTGGGTACTGGAGGCTATAGGACATTGTGTTTTTTGTCACCCGCAATTTCCCAATCCAACTGAATGGTGCCAGAATCTAATGATGTACAATTTGTCTTGCCTAGGATTGGCTCTCCATATGGCAGAACTTCTCCATCTAGAATACCATAGTCAGACCTGGTCCGAGGCTCAAATAGTTAATTACAGGCCCCACAACCCTAGGGCATATTCCTATAATGGAAAGTGGGGTGGGGGAGGAGGCCCGGGTCCTGAGGGCATTGTTCCGGCCTCGGCCATGCGGCCAGCCATTACCTTCTTTAAGATTGTTGTGCTTAGAGGCCCCTCCCAAGGAGTTTATCCTCCTAGGGCAGTTTTATGGTCAGGCCTATGTTAATTGAACTCTTGCTAGGGAATATTGGCACCCATCACCCAAGCCTCACTGGCCTAGTTCTTCAAGGACACGCTGGTACAGGGCCACTAATCCCAGCAGTCCCCATGTCCTTATGGTGGCCAATAAGGACATTAGACAGTGAGGGTGTGCCCATGATGCTGGTAAGCTTGATTAAACCCGGCACATGGGGGGGCCCAGAAGATGATCACGCATCTAGGGCACAAGGAAATTACAAGGTGCTATAACTGGGGGATGCCCCAGGGCTAGCAGTTGCAAGGAATAACCAGGGATGCGGGCATCCCTGTGTTCAATTTCAGATGGGTGCTACTCTTCCCAAGGCAAAATCACCCTTAAAGTGTATCCTGGATAACTGGGACCAATTTGACCCACAATCTTTGAAGAAGAAGCGGCTCATTTTTCTCTGCCAAACAGCATGGCCACAATACCCTCTGCTGAGTGGAGAAACCTGGCCTCCTAAGGGAAGTATAAATTATAACACCCTTCTACAATTAGACCTGTTCTGTAAGAGGGAAGGTAAATGGATTGAGGTCCCCTATGTGCAAGTTTTCTTTTCTCTAAGGAATAATCCTCAGCTATGTAAGGCCTGTAACCTATACCCTACTGGCCCCTCCTCAGGACTACCCCCATATTTAGGACTCCCTGTGGCTCCTCCCTCCACCGATGCTGACCAGGTCTCCTCAGCTCCTATAACCCAAGAGGAGTCAGGGAAGGCCAAAATGGCCAAGGCACCTCAGGCTACTAGGATGCCCCAACTCTGCCCCCTACAGGCAGTAGGAGGAGAATTTGGCCCAACCAAGGTACATACTCCCTTTTCACTTTCTGACTTAAAGCAAATCAAGGTGGACTTAGGAAAATTTTCAGATGACCCGGACAAGTACATAGATGTTCTGCAGGGCCTGGGTCAGTCCTTTGAATTAGATTGGAAAGATATCATGTTATTGCTTAGTCAGACCCTAACCAGCAATGAGAGAGAGGCTGCTCTAGCAGTGGCTCAAGAGTTTGGGGACACCTGGTACCTTAGTCAAGTACATGACCAAATGACGCCAGAGGAAAAGGATAGATTCCCTACAGGTAGACAAGCAGTCCCTAGCATGGACCCTCACTGGGATCCTGACTCAGAACAGGGGGACTGGAGTCATAGGCACCTATTGACCTGCATACTTGAGGGATTAAGGCGAACTAGGAAAAAGCCCATGAACTATGCAATGTTATCCACCATAACTCAGGGAAAGGAAGAAAACCCAACAGCCTTCCTTGAGTGGTTACGGGAGGCCTTAAGAAAGTATACTCCCCTGTCACCAGACTCCATCGAGGGTCAGTTAATACTAAAAGACAAATTTATTATGCAATCAGCAGCAGACATTAGGAGGAAGCTCCAGAAGCTGGCCCTGGGGCCAGAACAGAGTCTAGAATCATTACTAAATCTAGCAACCTCGGTGTTCTATAATAGAGACCAGGAGGAACAGGCTGAAAGGAACAGGAGAGACCAGAGAAAGGCCGCAGCCCTAGTCATGGCCTTCAGGCAAGCAGACCCAAGGGGCTCAGAAGAGGGAAAGGCTTGGGTTAGCCGCCAGTCTGGCAGAGCTTGCTACCACTGTGGCCTACCGGGACACTTTAAGAGAGATTGTCCCCAGAGGAATGGACCACCTCCTCGTCCTTGCCCACTGTGCCAAGGGGATCACTGGAAGGCACACTGTCCCAGGGGATGAAGGTTCCCAGGGCCTGAGGTGGCTAACCAGATGACCCAACAGCAGGACTGAGGGTGCCCGGGGCAAGTGCCAGCACAAGCCATCACCCTCACCGAGCCCCGGGTAAAGCTAACCATAGAGGGCCAGGAGGTTAATCTTCTCCTGGACACTGGCGCGGCCTTCTCAGTTTTGCTTTCCTGTCCTGGACGGTTGTCCTCCAAGTCTGTAACTGTCCGAGGAGTCCTAGGACAAGCAGTCTCCCGATATTTCTCCCAGCCCCTAAGCTGTAACTGGGGAGATATACTCTTCTCCCATGCCTTCCTCATCATGCCAGAAAGTCCCACTCCCTTACTAGGGTGGGACCTGTTAGCCAGAGCAGGAGCCATTATCTATATGAACATCATCAGGGAGGAAATCCCTCTCTGCTGCCCATTATTTAAAGAAGGGATTAATCCCGAGGTTTGGGCAATGGAGGGACAATTTGGACGGGCAAAGAATGCATGCCCAATTCGAATAAGGCTAAAGGATCCCACCTCTTTTCCTTACCAAAGACAGTACCCCCTAAGACCGGAAGCCAAAAAGGGGCTACAGGATATTGTAAGGAACTTAAAAGCTCAGGGTCTAGTAAAGTCCTGTAACAGCCCTTGTAATACTCCAATTTTAGGAGTACAAAAACCTAATGGACAATGGAGGCTGGTGCAAGATCTCAGGCTTATTAATGAAGCTGTGATCCCTTTATACCCAGCTGTACCCAACCCATACACCTTGCTCTCTCAAATACCAGGAGAGGCACAATGGTTTACAGTCCTGGACCTTAAGGATGCCTTTTTCTGTGTTCCATTACATCCTGACTCCCAGTTTCTGTTCGCCTTTGAAGACCCTTTAGACCAGAATTCTCAGCTCACCTGGACAGTGTTACCCCAGGGCTTTCGAGATAGCCCCCACCTGTTTAGCCAGGCTCTAGCTCAGGACCTTAGTCATTTTTCACATCCAGGTACCCTGGTTCTTCAATATATGGATGACTTACTTTTAACTGCTAACTCAGAAACCCAGTGTCAGCAGGCCACCCAGGACCTCCTAAACTTTCTAGCCACCTGTGGATATAAGGTTTCCAAACCAAAAGCCCAACTTTGTAGGCAAGAGGTAAAATACCTAGGGCTTGTCTTGTCTAAGGGCACTCGGGCCCTTGATGGAGAACGTCTCCAACCCATACTGGCCTTCCCCCATCCAAAGACCTTAAAACAGTTGTGGGGATTCTTAGGAATAACTGGTTTCTGCCGATTGTGGATTCCCAGATATGGTGAAATAGCCAAACCCCTATACACTCTGATTAAGGAAACCCAAAAAGCTAACACCCATCTAATACAATGGGACCCTATAGCTGAGGCAGCCTTTCAGACCCTGAAGCAGGCACTACTGCAGGCTCCAGCTCTAAGCCTTCCTACAGGGAACGACTTCTCCCTATATGTTACGGAAAAGTCAGGAGTGGCACTGGGGGTTCTCACCCAGACTCGGGGGACCACGCCACAACCGGTGGCATACCTAAGTAAGGAAATTGATGCAGTAGCAAAAGGCTGGCCACACTGCTTGCGGGTCATAGCCACAGTGGCTATACTGGTCTCAGAAGCTGTTAAAATAGTGCAAGGGAAAGATCTAACTGTATGGACTTCACATGATGTAGCTGGAATTCTGGCCTCTAAAGGAGGCTTATGGCTGTCTGACAACCGCTTACTCAAATATCAGGCCTTAATGCTCGAAGGGCCTGTACTTCAGCTGCGCACATGTGCAGCCCTCAATCCAGCTACTTTTCTTCCTGAGGAAGGGGCAGACAAAGAACATGACTGTCAGCAGGTTATAGCCCAGAACTATGCGGCTCAAGAGGATCTCTTAGAAACTCCTCTAGCCAATCCTGACCTTAACCTATACACTGACGGAAGTTCATTTATGGAAAATGGAGTTCGAAAGGCAGGATATGCAGTAGTCAGTGATCAAACAGTGCTTGAAAGTAATTCCCTTCCTCCTGGAACTAGTGCCCAGCTGGCAGAACTAGTAGCCCTCACTCAAGCTCTAAAATTAGGAAAAGGAAAGAGAATAAACATATACACAGATTCCAAATATGCTTACCTGGTTCTGCATGCCCATGCTGCCATATGGAAGGAAAGAGAGTTCCTAACCTCGGCAGGAACTCCCATTAAATATCATAAGGAAATTTTAGACTTATTACAAGCTGTCCAAGAACCCAAAGAGGTAGCAGTCTTACACTGCCGAGGTCACCAGAAAGGAGATGAGAAGGAAGCAGAGGGCAATCGCCGAGCTGACCTAGAGGCAAAAAGGGCAGCTAGACAAGAATTTATAGAAGGACCTTTAATATGGGAAAACCCTCTCCAGGGAACTAGACCTCAATACTCATCAGAAGAGGTAGAGTGGGCAATCTCCCAGGGACATAATCTTCTTCCTTCAGGATGGCTAGCTACTGAGGAGGGAAAAGTACTCCTACCTGCTGCCAGCCAGTGGAAAGTACTTAAAACCCTACATCAAACCTTCCATATGGGTATAGAGAATACACATCAAATGGCCAAATCTATATTTACAGAAAAAGGTCTCTTCAAGGCTGTCCAACAAATAGTCAAAGGATGTGAAATATGCCAGAAAAATAATCCTTTGGCACATCGCAAAGCTCCTCCTGGGGAACAGCGAACCGGTCATTACCCAGGTGAGGACTGGCAAATGGACTTTACCCATATGCCTAAGTCTCAGGGATATCAATATCTGTTAGTATGTGTAGATACCTACACAAACTGGGTTGAGGCTTTCCCCTGTAGAACAGAAAAAGCCCAAGAGGTAATAAAAGTCCTGATTAATGAAATAATCCCTAGATTCAGACTCCCTCTGTCTTCAAAGTGATAACGGCCCTGCCTTCAAAGCTGCAGTGACCCAAGGAATTTCCAAGGCGTTAGGAATACAGTATCATCTCCACTGTGCTTGGAGGCCTCAGTCCTCAGGGAAAGTGGAAAAGATGAATGAAATTCTTAAGAGACACCTAAAGAAACTGGCACAGGAAACCCACCTCCCGTGGCCTTCTCTATTACCAATAGCACTCTTAAGGGCTCGAAACTCTCCTCAGAAAATAGGACTCAGCCCCTATGAGATGCTTTATGGATGGCCCTTTCTTACTAATGATCTTGTATTAGACAAGGAAACAGCAGATCTAATAAAAGATATAACCTCCTTGGCTAAGTACCAACAGGCTCTTAGGACCTTACCTGAAGGACACCCTAGGGAAAAAGGGAAAGAGTTGTTCTGCCCTGGAGACTTGGTGCTTGTTAAGTCTCTCCCCTCAGACTCTCCCTCTCTAGGCCCATCTTGGGAAGGACCATTTTCAGTTATTCTGTCTACCCCAACGGCAGTTAAGGTGGTGGGAATTGACTCATGGATTCATCACACTCGGCTGAAGCCTTGGACACCTCCTGAAGAAAATCATAGGCCCTCCACTCCACAGCCTGAAGTTCCAGGAGACGACCCTTCATACACCTGCGAGCCATTAGAGGATCTACATTTGCTCTTCCAACAAGATCCCCAGGAAAGTAACTACTCATGAGTCTAACTTACATCCTGGCATTAACAGGACTATCACTCTTAACTTTACTATCTGCAACCGGACTCTATGTGGTTGCCCCTACGGATTGGCCTAATAGCTCTCCTCCTCTCCTTCTGATATTATCACTCCCTTACTATGACCTCTCTTGTGGTCTTCATTATCCTCTTTGCTGCCTGTCCCCTGCTTTATTCCCATCCCACCTGCAAATGCACCCAAAGGGGTTACAACTGGAACAATATTGGACAGGGAACAAGTCCATTCTCTGGTTGGTCTTGGGGATTAGGTCGACAAGCCTCCTTTATATCCTATACTTATATGCACAAAAATTGCTATACATCTGCTTCCCTGTGCACATACAACTCACACACATATACTGGACAGGAACAATAACCAGGCCTAATTGTCCAGCTGCTAAGAAAACCACCACCTGCTGGACATATTTTACCCACGTCGGGACTTCAGATGAGGGCAAAGTTCAACATCAGGCACAGAAACAGCATCTCAAAGAAGTAATCTCTAGCCTAGGCAAACCACAAAGTACCCCCAGTCCCTACAAGGGCTTGGACCTTTCTAAGCTACATGAGACCCTTCACACACACTCCTGTATGCTCAACTTATTTAACACCACACTCACAGGAATGCATGAGGCCTCCTCCAACAACCCAACTGATTGTTGGATGTGCCTCCCACTACGCTTCAAAGCATATATCCCGGTTTCATTCAATACTCATGGAACACAACTAACCCACCCTCAAATCATACGGGGCTTATAGGTCCCTTGGTCACCAATCTACAAATTCCATCTACCTCAAACTTCACTTGTGTGAACTCTACTAACAACTCCAATGCTTCCCTATGCCAAAACTGGGTTGCCTTACCCCCAGGCCTCAACTGCCTAGCACCAGGCTTATTCTTTGTTTGTGGCACCTCAGCTTACCGCTGCCTGAACAATACCCCTGGGGAAATATGCCTCCTCTCCTTTCCTAGTGCCCCCCCCATGACGATTTACACCGACTCTCAGGTGCAGAGTCTTCTCTTGCCAAGGCCACGCCATACTCAAACTCCAATTATTCCTTTCATAGTGTGGGGGGGGCCGGAATAATAGGAGGACTTGGTACTGGCATTGGAAAAGTTACTACCTCTACTCAGTTTTACTATAAGCTATCTCAGGAGTTAAATAATAACATGGAAAAGGTTGCTAATTCTCTCTGCAAAGGCAGCTCACTCTGCAGAAGCAGCTTAACTCTTTAGCTGCCATAGCTCTTCAAAATAGAAGGGCCCTGGATCTGCTAACAGCAGAGAGAGAAGGAACCTGTCTCTTTTTAGGAAAAAAATGCTGCTACTTTGTAAACCAATCAGGTATTGTCTTAAACAAAGTTAAAGAACTCAGGGATCAAATCAAACACAGGGCTAAAGACCTTCAAGAAACAGGATCATGGAGCTTGTTTAACAACTGGCTGTCCTGGCTCTACCCTCTCCTAGGCCCGTTTATAACCATTCTATTATTGCTGGCCTTTGGGCCTCTCATCTTTAACCTTCTTGTCAAATTCATTTCTTCTAGAATTAAAGCCATAAGGCCACAAATGATCCTGCAAATGGAACCCCGGATGTCCTTGGCTCATGAGTTCTATCGTGGACCTCTGGATCAACCTGCACCCCTGGAAGACTCCCCTCTGGAGGAAACCTCAACTGCAGGGCCCCTTCTATGCCCCAATTCAGCAGGAAGTAGTTAAAGAGCGATCGACGCCCGTGTCCCAACAGCAGTTGGACTTTCCTGTTCAGAGGGGGGACTGAGGGACTGTGTCTCTTCCCGGGTCCACAAGGGGCGTGGTTTCAAAGGCAAGAATTTCCCAGCTCCATGCCTCCATCCAGGTCCCAGAGGGGTTTCAAATGTAAAGTTCTTTGTCTAACTTTCCCGCGCGCGGGCTAGGTAGAGAAATCCAGGTGAGCTTCCTTTGTTCAAAAAGCCCACCAAAAGCTCTGGGAGGAGGGGGCTCTGTTGTGGACCAAGCAGCCAATCCAGTATCAGAGTCAAAGATCAATCACTCCAGAGGAAGTTTGAAAGAACTCCTCTCATTGGACGAGAACATGAATGGGGAGGGAATAACTCCAGGGTTAAAATTCCACTGCCCCTTACCTATATGGATTTCCCCTCTGGGWTCCCCTCCCACAACAGCGTGGGAGCTGTCTCTCTCTCTCTCTCTCTCTCTCTCTCTGCCTAATAAATTCCTTTTCTGCAAAACTCTTTGGGTCCTGATATTTATTTGAACGGTAAGCTCACCGCACCTCCAGGGCCCCTTTCCCCATTCTTGGGGGTAAGAGAGGCCAAGAACCTGGAAAATCTGGGAACTGAGAGCATCCTCCTCCAGTTACA

>ERV1-1_CalJac_PVconsensus_LTR

TGAGGGACTGTGTCTCTTCCCGGGTCCACAAGGGGCGTGGTTTCAAAGGCAAGAATTTCCCAGCTCCATGCCTCCATCCAGGTCCCAGAGGGGTTTCAAATGTAAAGTTCTTTGTCTAACTTTCCCGCGCGCGGGCTAGGTAGAGAAATCCAGGTGAGCTTCCTTTGTTCAAAAAGCCCACCAAAAGCTCTGGGAGGAGGGGGCTCTGTTGTGGACCAAGCAGCCAATCCAGTATCAGAGTCAAAGATCAATCACTCCAGAGGAAGTTTGAAAGAACTCCTCTCATTGGACGAGAACATGAATGGGGAGGGAATAACTCCAGGGTTAAAATTCCACTGCCCCTTACCTATATGGATTTCCCCTCTGGGWTCCCCTCCCACAACAGCGTGGGAGCTGTCTCTCTCTCTCTCTCTCTCTCTCTCTCTGCCTAATAAATTCCTTTTCTGCAAAACTCTTTGGGTCCTGATATTTATTTGAACGGTAAGCTCACCGCACCTCCAGGGCCCCTTTCCCCATTCTTGGGGGTAAGAGAGGCCAAGAACCTGGAAAATCTGGGAACTGAGAGCATCCTCCTCCAGTTACA

>ERV1-1_CalJac_PVconsensus_INTERNAL

TCTTTGGCGAGCCAGCCAGGAGACATCAGACTTCAGAGTGGTGAGTGATATTGGACCCCTTTCACTTGCTAATCTGTCCTTTCCCTTCTTTTAAATTTGTTAAGGAGGTAAAGACTGGGCACCTGTCAGCCATCTAAGAGCGACTAGCGTAGCCACCAGGCTAAAGACACGGGTGTCAGGCTTTCTGGGAACGGGCTCTCTAGCAACCCCCAGCTTTTCAGAACTGGGAGCATTGGTTAGCCCTGAACCAGCTTCCCTTTCTTGCTGCTCTTTCTTTCTCTGGGCCAAGCCGAGGGCCGGCAGGAGGAAAGGCGGTCCCAAACTCCCTGTTCAGTCCAGTTGAGGTTACGGCCCTGCTTAGACCTCCTAAGAAAAAGCCGCCTATGCGAGCCATGCCCAGCCTTTCAGACCCCAACCTCCTGGGTCATTTCCATGAGGCAAACTTCTTCCCGGCTCTTTCCTCTAAGATATTTCCTACACATGAGGCTATTACCCTTCTTGCTAGGACTTTGAGGCTCTTGGGGTGGCAGTCCAGGGACCCTTGCCCATGGTGCCCTCAGGCTTATGCCTATGAGGACCTCCTACAGTATAAATGCACAGGGCCAGTTCCCTTAGTCTGCAGTGCTAGGTTATGTCCTAACAGAGCCAGAGTCTATCAGTGGGTACTGGAGGCTATAGGACATTGTGTTTTTTGTCACCCGCAATTTCCCAATCCAACTGAATGGTGCCAGAATCTAATGATGTACAATTTGTCTTGCCTAGGATTGGCTCTCCATATGGCAGAACTTCTCCATCTAGAATACCATAGTCAGACCTGGTCCGAGGCTCAAATAGTTAATTACAGGCCCCACAACCCTAGGGCATATTCCTATAATGGAAAGTGGGGTGGGGGAGGAGGCCCGGGTCCTGAGGGCATTGTTCCGGCCTCGGCCATGCGGCCAGCCATTACCTTCTTTAAGATTGTTGTGCTTAGAGGCCCCTCCCAAGGAGTTTATCCTCCTAGGGCAGTTTTATGGTCAGGCCTATGTTAATTGAACTCTTGCTAGGGAATATTGGCACCCATCACCCAAGCCTCACTGGCCTAGTTCTTCAAGGACACGCTGGTACAGGGCCACTAATCCCAGCAGTCCCCATGTCCTTATGGTGGCCAATAAGGACATTAGACAGTGAGGGTGTGCCCATGATGCTGGTAAGCTTGATTAAACCCGGCACATGGGGGGGCCCAGAAGATGATCACGCATCTAGGGCACAAGGAAATTACAAGGTGCTATAACTGGGGGATGCCCCAGGGCTAGCAGTTGCAAGGAATAACCAGGGATGCGGGCATCCCTGTGTTCAATTTCAGATGGGTGCTACTCTTCCCAAGGCAAAATCACCCTTAAAGTGTATCCTGGATAACTGGGACCAATTTGACCCACAATCTTTGAAGAAGAAGCGGCTCATTTTTCTCTGCCAAACAGCATGGCCACAATACCCTCTGCTGAGTGGAGAAACCTGGCCTCCTAAGGGAAGTATAAATTATAACACCCTTCTACAATTAGACCTGTTCTGTAAGAGGGAAGGTAAATGGATTGAGGTCCCCTATGTGCAAGTTTTCTTTTCTCTAAGGAATAATCCTCAGCTATGTAAGGCCTGTAACCTATACCCTACTGGCCCCTCCTCAGGACTACCCCCATATTTAGGACTCCCTGTGGCTCCTCCCTCCACCGATGCTGACCAGGTCTCCTCAGCTCCTATAACCCAAGAGGAGTCAGGGAAGGCCAAAATGGCCAAGGCACCTCAGGCTACTAGGATGCCCCAACTCTGCCCCCTACAGGCAGTAGGAGGAGAATTTGGCCCAACCAAGGTACATACTCCCTTTTCACTTTCTGACTTAAAGCAAATCAAGGTGGACTTAGGAAAATTTTCAGATGACCCGGACAAGTACATAGATGTTCTGCAGGGCCTGGGTCAGTCCTTTGAATTAGATTGGAAAGATATCATGTTATTGCTTAGTCAGACCCTAACCAGCAATGAGAGAGAGGCTGCTCTAGCAGTGGCTCAAGAGTTTGGGGACACCTGGTACCTTAGTCAAGTACATGACCAAATGACGCCAGAGGAAAAGGATAGATTCCCTACAGGTAGACAAGCAGTCCCTAGCATGGACCCTCACTGGGATCCTGACTCAGAACAGGGGGACTGGAGTCATAGGCACCTATTGACCTGCATACTTGAGGGATTAAGGCGAACTAGGAAAAAGCCCATGAACTATGCAATGTTATCCACCATAACTCAGGGAAAGGAAGAAAACCCAACAGCCTTCCTTGAGTGGTTACGGGAGGCCTTAAGAAAGTATACTCCCCTGTCACCAGACTCCATCGAGGGTCAGTTAATACTAAAAGACAAATTTATTATGCAATCAGCAGCAGACATTAGGAGGAAGCTCCAGAAGCTGGCCCTGGGGCCAGAACAGAGTCTAGAATCATTACTAAATCTAGCAACCTCGGTGTTCTATAATAGAGACCAGGAGGAACAGGCTGAAAGGAACAGGAGAGACCAGAGAAAGGCCGCAGCCCTAGTCATGGCCTTCAGGCAAGCAGACCCAAGGGGCTCAGAAGAGGGAAAGGCTTGGGTTAGCCGCCAGTCTGGCAGAGCTTGCTACCACTGTGGCCTACCGGGACACTTTAAGAGAGATTGTCCCCAGAGGAATGGACCACCTCCTCGTCCTTGCCCACTGTGCCAAGGGGATCACTGGAAGGCACACTGTCCCAGGGGATGAAGGTTCCCAGGGCCTGAGGTGGCTAACCAGATGACCCAACAGCAGGACTGAGGGTGCCCGGGGCAAGTGCCAGCACAAGCCATCACCCTCACCGAGCCCCGGGTAAAGCTAACCATAGAGGGCCAGGAGGTTAATCTTCTCCTGGACACTGGCGCGGCCTTCTCAGTTTTGCTTTCCTGTCCTGGACGGTTGTCCTCCAAGTCTGTAACTGTCCGAGGAGTCCTAGGACAAGCAGTCTCCCGATATTTCTCCCAGCCCCTAAGCTGTAACTGGGGAGATATACTCTTCTCCCATGCCTTCCTCATCATGCCAGAAAGTCCCACTCCCTTACTAGGGTGGGACCTGTTAGCCAGAGCAGGAGCCATTATCTATATGAACATCATCAGGGAGGAAATCCCTCTCTGCTGCCCATTATTTAAAGAAGGGATTAATCCCGAGGTTTGGGCAATGGAGGGACAATTTGGACGGGCAAAGAATGCATGCCCAATTCGAATAAGGCTAAAGGATCCCACCTCTTTTCCTTACCAAAGACAGTACCCCCTAAGACCGGAAGCCAAAAAGGGGCTACAGGATATTGTAAGGAACTTAAAAGCTCAGGGTCTAGTAAAGTCCTGTAACAGCCCTTGTAATACTCCAATTTTAGGAGTACAAAAACCTAATGGACAATGGAGGCTGGTGCAAGATCTCAGGCTTATTAATGAAGCTGTGATCCCTTTATACCCAGCTGTACCCAACCCATACACCTTGCTCTCTCAAATACCAGGAGAGGCACAATGGTTTACAGTCCTGGACCTTAAGGATGCCTTTTTCTGTGTTCCATTACATCCTGACTCCCAGTTTCTGTTCGCCTTTGAAGACCCTTTAGACCAGAATTCTCAGCTCACCTGGACAGTGTTACCCCAGGGCTTTCGAGATAGCCCCCACCTGTTTAGCCAGGCTCTAGCTCAGGACCTTAGTCATTTTTCACATCCAGGTACCCTGGTTCTTCAATATATGGATGACTTACTTTTAACTGCTAACTCAGAAACCCAGTGTCAGCAGGCCACCCAGGACCTCCTAAACTTTCTAGCCACCTGTGGATATAAGGTTTCCAAACCAAAAGCCCAACTTTGTAGGCAAGAGGTAAAATACCTAGGGCTTGTCTTGTCTAAGGGCACTCGGGCCCTTGATGGAGAACGTCTCCAACCCATACTGGCCTTCCCCCATCCAAAGACCTTAAAACAGTTGTGGGGATTCTTAGGAATAACTGGTTTCTGCCGATTGTGGATTCCCAGATATGGTGAAATAGCCAAACCCCTATACACTCTGATTAAGGAAACCCAAAAAGCTAACACCCATCTAATACAATGGGACCCTATAGCTGAGGCAGCCTTTCAGACCCTGAAGCAGGCACTACTGCAGGCTCCAGCTCTAAGCCTTCCTACAGGGAACGACTTCTCCCTATATGTTACGGAAAAGTCAGGAGTGGCACTGGGGGTTCTCACCCAGACTCGGGGGACCACGCCACAACCGGTGGCATACCTAAGTAAGGAAATTGATGCAGTAGCAAAAGGCTGGCCACACTGCTTGCGGGTCATAGCCACAGTGGCTATACTGGTCTCAGAAGCTGTTAAAATAGTGCAAGGGAAAGATCTAACTGTATGGACTTCACATGATGTAGCTGGAATTCTGGCCTCTAAAGGAGGCTTATGGCTGTCTGACAACCGCTTACTCAAATATCAGGCCTTAATGCTCGAAGGGCCTGTACTTCAGCTGCGCACATGTGCAGCCCTCAATCCAGCTACTTTTCTTCCTGAGGAAGGGGCAGACAAAGAACATGACTGTCAGCAGGTTATAGCCCAGAACTATGCGGCTCAAGAGGATCTCTTAGAAACTCCTCTAGCCAATCCTGACCTTAACCTATACACTGACGGAAGTTCATTTATGGAAAATGGAGTTCGAAAGGCAGGATATGCAGTAGTCAGTGATCAAACAGTGCTTGAAAGTAATTCCCTTCCTCCTGGAACTAGTGCCCAGCTGGCAGAACTAGTAGCCCTCACTCAAGCTCTAAAATTAGGAAAAGGAAAGAGAATAAACATATACACAGATTCCAAATATGCTTACCTGGTTCTGCATGCCCATGCTGCCATATGGAAGGAAAGAGAGTTCCTAACCTCGGCAGGAACTCCCATTAAATATCATAAGGAAATTTTAGACTTATTACAAGCTGTCCAAGAACCCAAAGAGGTAGCAGTCTTACACTGCCGAGGTCACCAGAAAGGAGATGAGAAGGAAGCAGAGGGCAATCGCCGAGCTGACCTAGAGGCAAAAAGGGCAGCTAGACAAGAATTTATAGAAGGACCTTTAATATGGGAAAACCCTCTCCAGGGAACTAGACCTCAATACTCATCAGAAGAGGTAGAGTGGGCAATCTCCCAGGGACATAATCTTCTTCCTTCAGGATGGCTAGCTACTGAGGAGGGAAAAGTACTCCTACCTGCTGCCAGCCAGTGGAAAGTACTTAAAACCCTACATCAAACCTTCCATATGGGTATAGAGAATACACATCAAATGGCCAAATCTATATTTACAGAAAAAGGTCTCTTCAAGGCTGTCCAACAAATAGTCAAAGGATGTGAAATATGCCAGAAAAATAATCCTTTGGCACATCGCAAAGCTCCTCCTGGGGAACAGCGAACCGGTCATTACCCAGGTGAGGACTGGCAAATGGACTTTACCCATATGCCTAAGTCTCAGGGATATCAATATCTGTTAGTATGTGTAGATACCTACACAAACTGGGTTGAGGCTTTCCCCTGTAGAACAGAAAAAGCCCAAGAGGTAATAAAAGTCCTGATTAATGAAATAATCCCTAGATTCAGACTCCCTCTGTCTTCAAAGTGATAACGGCCCTGCCTTCAAAGCTGCAGTGACCCAAGGAATTTCCAAGGCGTTAGGAATACAGTATCATCTCCACTGTGCTTGGAGGCCTCAGTCCTCAGGGAAAGTGGAAAAGATGAATGAAATTCTTAAGAGACACCTAAAGAAACTGGCACAGGAAACCCACCTCCCGTGGCCTTCTCTATTACCAATAGCACTCTTAAGGGCTCGAAACTCTCCTCAGAAAATAGGACTCAGCCCCTATGAGATGCTTTATGGATGGCCCTTTCTTACTAATGATCTTGTATTAGACAAGGAAACAGCAGATCTAATAAAAGATATAACCTCCTTGGCTAAGTACCAACAGGCTCTTAGGACCTTACCTGAAGGACACCCTAGGGAAAAAGGGAAAGAGTTGTTCTGCCCTGGAGACTTGGTGCTTGTTAAGTCTCTCCCCTCAGACTCTCCCTCTCTAGGCCCATCTTGGGAAGGACCATTTTCAGTTATTCTGTCTACCCCAACGGCAGTTAAGGTGGTGGGAATTGACTCATGGATTCATCACACTCGGCTGAAGCCTTGGACACCTCCTGAAGAAAATCATAGGCCCTCCACTCCACAGCCTGAAGTTCCAGGAGACGACCCTTCATACACCTGCGAGCCATTAGAGGATCTACATTTGCTCTTCCAACAAGATCCCCAGGAAAGTAACTACTCATGAGTCTAACTTACATCCTGGCATTAACAGGACTATCACTCTTAACTTTACTATCTGCAACCGGACTCTATGTGGTTGCCCCTACGGATTGGCCTAATAGCTCTCCTCCTCTCCTTCTGATATTATCACTCCCTTACTATGACCTCTCTTGTGGTCTTCATTATCCTCTTTGCTGCCTGTCCCCTGCTTTATTCCCATCCCACCTGCAAATGCACCCAAAGGGGTTACAACTGGAACAATATTGGACAGGGAACAAGTCCATTCTCTGGTTGGTCTTGGGGATTAGGTCGACAAGCCTCCTTTATATCCTATACTTATATGCACAAAAATTGCTATACATCTGCTTCCCTGTGCACATACAACTCACACACATATACTGGACAGGAACAATAACCAGGCCTAATTGTCCAGCTGCTAAGAAAACCACCACCTGCTGGACATATTTTACCCACGTCGGGACTTCAGATGAGGGCAAAGTTCAACATCAGGCACAGAAACAGCATCTCAAAGAAGTAATCTCTAGCCTAGGCAAACCACAAAGTACCCCCAGTCCCTACAAGGGCTTGGACCTTTCTAAGCTACATGAGACCCTTCACACACACTCCTGTATGCTCAACTTATTTAACACCACACTCACAGGAATGCATGAGGCCTCCTCCAACAACCCAACTGATTGTTGGATGTGCCTCCCACTACGCTTCAAAGCATATATCCCGGTTTCATTCAATACTCATGGAACACAACTAACCCACCCTCAAATCATACGGGGCTTATAGGTCCCTTGGTCACCAATCTACAAATTCCATCTACCTCAAACTTCACTTGTGTGAACTCTACTAACAACTCCAATGCTTCCCTATGCCAAAACTGGGTTGCCTTACCCCCAGGCCTCAACTGCCTAGCACCAGGCTTATTCTTTGTTTGTGGCACCTCAGCTTACCGCTGCCTGAACAATACCCCTGGGGAAATATGCCTCCTCTCCTTTCCTAGTGCCCCCCCCATGACGATTTACACCGACTCTCAGGTGCAGAGTCTTCTCTTGCCAAGGCCACGCCATACTCAAACTCCAATTATTCCTTTCATAGTGTGGGGGGGGCCGGAATAATAGGAGGACTTGGTACTGGCATTGGAAAAGTTACTACCTCTACTCAGTTTTACTATAAGCTATCTCAGGAGTTAAATAATAACATGGAAAAGGTTGCTAATTCTCTCTGCAAAGGCAGCTCACTCTGCAGAAGCAGCTTAACTCTTTAGCTGCCATAGCTCTTCAAAATAGAAGGGCCCTGGATCTGCTAACAGCAGAGAGAGAAGGAACCTGTCTCTTTTTAGGAAAAAAATGCTGCTACTTTGTAAACCAATCAGGTATTGTCTTAAACAAAGTTAAAGAACTCAGGGATCAAATCAAACACAGGGCTAAAGACCTTCAAGAAACAGGATCATGGAGCTTGTTTAACAACTGGCTGTCCTGGCTCTACCCTCTCCTAGGCCCGTTTATAACCATTCTATTATTGCTGGCCTTTGGGCCTCTCATCTTTAACCTTCTTGTCAAATTCATTTCTTCTAGAATTAAAGCCATAAGGCCACAAATGATCCTGCAAATGGAACCCCGGATGTCCTTGGCTCATGAGTTCTATCGTGGACCTCTGGATCAACCTGCACCCCTGGAAGACTCCCCTCTGGAGGAAACCTCAACTGCAGGGCCCCTTCTATGCCCCAATTCAGCAGGAAGTAGTTAAAGAGCGATCGACGCCCGTGTCCCAACAGCAGTTGGACTTTCCTGTTCAGAGGGGGGAC

>ERV1-1_SaiBol_PVconsensus

TGAGGGACTGTGTTTCTTCCCGGGTCCACAAGGGGCGTGGTTTCAAAGGCAAGAATTTCCCAGCGCCATGCCTCCACTCCTCCATCTGGGAACCTAAAGTTCTTTGTCTAAMTTTCCCGCGCGCGGGCTAGGTAGARAAATCCAGGTGAGGCTCCTTTGTCCCAAAAAGCCCGCCGAAAGCTCTGGGAGGAGGGGGCTCTGTTGTGGACCAAGCAGCCAATCCAGTATCAGAGTCAAAGATCAATCACTCCAGAGGAAGTTTGAAAGAACTCCTCTCATTGGACGAGAACATGAATGGGGAGGGAATAACTCCAGGGTTAAAAGTCCCCCTGCTCCTTACCTATATGGATCTCCATCTCTGGATCCCCTCCCACGACAGCGTGGGAGCTGTCTCTCTCTCTTTCTCTCTCTCTGCCTAATAAATCGCTTTTCTGCAAAACTCTTTGGGTTCATGATATTTACTTGAACGGTAAGCTCACCGCGCCTCCGGGGCCCCTTCCCCCATTCTTGGGGGTRAGAGAGGCCAAGAACCTGGAATTAAGATCGGGGAGCTGGGAGTGCAYCCGACCCCAGTTACATCTTTGGCGAGCCAGCCAGGAGACATCAGACTTCAGAGTGGTGAGTGATATTGGACCCCTTTCACTTGCTAATCTGTCCTTTCTCTTCTTTTAAATTTGTTAAGGAGATAAAGACCGGGCGCCTGTTGGCCATCTAAGAGCAACTAGCGCGGCCACCGGCTAAAGACACGGGTGTCAGGCTTTCTGGGAACGGACTCTCCAGCAACCCCCAGCTTTTCAGAACTGGGAGCGTTGGTCTAGCCCCGAACCAGCTTCCCTTTCTTGCCTGCTCTTTCTTTCTCTGGGCCAAGCCGAGGGCCGGCAGGAGGAAAGGCAATGGTCCTAAACTCCCGGTTCAGTCCAGTTGAGGTTATGGCCCTACTTAGACCTCCTAAGAAAAAGCCGCCTGTGTGAGCCACGCCCAGCCTTTCAGACCCCAACCTCCTGGGTCCAGGCAATTTCCATGAGGCAAACTTCCCGGCTCTTTCCTCTAAGATATTTCCTACACATGAGGCTATTACCCTTCTTGCTAGGACTTTGAGGCTCTTGGGGTGGCAGTCCAGGGACCCTTGCCCGTGGTGCCCTCAGGCTTATGCCTATGAGGACCTCCTACAGTATAAATGCACAGGGCCAGTTCCCTTAGTCCGCAGTGCTAGGTTATGTCCTAACAGAGCCAGAGTCTATCAGTGGAACCTTTTGGGGAAATCCAGGTACTGGAGGCTATAGGACATTGTGTTTTTTGTCACCCGCAATTTCCCAATCCAACTGAATGGTGCCAGAATCTAATGATGTACAATTTGTCTTGCCTAGGATTGGCTCTCCCTACGGCAGAACTTCTCCATCTAGAATACCATAGTCAGACCTGGTCCAAGGCTCAAATAGTTAATTACAGGCCCCACAACCCTAGGGCATATTCCTATAATGGAAAGTGGGGTGGGGGAGGAGGCCCGGGTCCTGAGGGCATTGTTCCAGCCTCAGCCATGTGGCCAGCCATTACCTTCTTTAAGATTGTTGTGCTTAGAGGCCCCTCCCAAGAAGTTTATCCTCCTAGGGCAGTTTTATGATCAGGCCTATGTTAATTGAACTCTTGCTAGGGAATATTGGCACCCATCACCCAAGCCTCACTGGCCTAGTTCTTCAAGGACACGCTGGTACAGGACCACTAATCCCAGCAGTCCCCATGTCCTTATGGTGGTCAATAAGGACATGTAGACAGTGAGGGTGTGCCCATGACGCTGGTAAGCATGATTAAACCCAGCACATGGGAGGGGCCCAGAAGATGATCACGCATCTAGGGCACAAGGAAATTACAAGGTGCTATAACTGGGGGATGCCCCAGGGCTAGCAGTTGCAAGGAATAACCAGGGATGCGGGCATCCCTGTGTTCAATTTCAGATGGGTGCTACTCTTCCCAAGGCAAAATCACCCTTAAAGTGTATCCTGGATAACTGGGACCAATTTGACACACAATCTTTGAAGAAGAAGTGGCTCATTTTTCTCTGCCAAACAGCGTGGCCACAATACCCTCTGCTGAGTGGAGAAACCTGGCCTCCTAAGGGAAGTATAAATTATAACACCCTTCTACAATTAGACCTGTTCTGTAAGAGGGAAGGTAAATGGATTGAGGTCCCCTATGTGCAAGTTTTCTTTTCTCTAAGGGATAATCCTCAGCTATGTAAGGCCTGTAACCTATACCCTACTGGCCCCTCCTCAGGACTACCCCCATATTTAGGACTCCCTGTGGCTCCTCCCTCCACCAATGCTGACCAGGTCTCCTCAGCTCCTATAACCCAAGAGGAGTCAGGGAAGGCCAAAATGGCCAAGGCACCTCAGGCTACTAGGATGCCCCAACTCTGCCCCCTACAGGCAGTAGGAGGAGAATTTGGCCCAACCAAGGTACATACTCCCTTTTCACTTTCTGACTTAAAGCAAATCAAGGCGGACTTAGGAAAATTTTCAGATGACCCAGACAAGTACATAGATGTTCTGCAGGGCCTGGGTCAGTCCTTTGAATTAGATTGGAAAGATATCATGTTATTGCTTAGTCAGACCCTAACCAGCAATGAGAGAGAGGCTGCTCTAGCAGTGGCTCAAGAGTTTGGGGACACCTGGTACCTTAGTCAAGTACATGACCAAATGACGCCAGAGGAAAAGGATAGATTCCCTACAGGTAGACAAGCAGTCCCTAGCATGGACCCTCACTGGGATCCTGACTCAGAACAGGGGGACTGGAGTCGTAGGCACCTATTGACCTGCATACTTGAGGGATTAAGGCGAACTAGGAAAAAGCCCATGAACTATGCAATGTTATCCACCATAACTCAGGGAAAGGAAGAAAACCCAACAGCCTTCCTTGAGTGATTACGGGAGGCCTTAAGAAAGTATACTCCCCTGTCACCAGACTCCATCGAGGGTCAGTTAATACTAAAAGACAAATTTATTATGCAATCAGCAGCAGACATTAGGAGGAAGCTCCAGAAGCTGGCCCTGGGGCCAGAACAGAGTCTAGAATCATTACTAAATCTAGCAACCTCGGTGTTTTATAATAGAGACCAGGAGGAACAGGCTGAAAGGAACAGGAGAGACCAGAGAAAAGCCGCAGCCCTAGTCATGGCCTTCAGGCAAGCAGACCCAAGGGGCTCAGAAGAGGGAAAGGCTTGGGTTAGCCGCCAGTCTGGCAGAGCTTGCTACCACTGCGGCCTAACGGGACACTTTAAAAGAAATTGTCCCCAGAGGAATGGACCACCTCCTCGTCCTTGCCCACTGTGCCAAGGGGATCACTGGAAAGCACACTGTCCCAGGGGATGAAGGTTCCCAGGGCCTGAGGTGGCTAACCAGATGACCCAACAGCAGGACTGAGGGTGCCCGGGGCAAGTGCCAGCACAAGCCATCACCCTCACCGAGCCCCGGGTAAAGCTAACCATAGAGGGCCAGGAGGTTGATCTTCTCCTGGACACTGGCATGGCCTTCTCAGTTTTGCTTTCCTGTCCTGGACAGTTGTCCTCCAAGTCTGTAACTGTCCGAGGAGTCCTAGGACAAGCAGTCTCCCGATATTTCTCCCAGCCCCTAAGCTGTAACTGGGGAGATATACTCTTCTCCCATGCCTTCCTCATCATGCCAGAAAGTCCCACTCCCTTACTAGGGCGGGACCTGTTAGCCAGAGCAGGAGCCATTATCTATATGAACATCATCAGGGAGGAAATCCCTCTCTGCTGCCCATTATTTAAAGAAGGGATTAATCCTGAGGTTTGGGCAATGGAGGGACAATTTGGATGGGCAAAGAATGCATGCCCAATTCGAATAAGGCTAAAGGATCCCACCTCTTTTCCTTACCAAAGACAGTACCCCCTAAGACCGGAAGCCAAAAAGGGGCTACAGGATATTGTAAGGAACTTAAAAGCTCAGGGTCTAGTAAAGTCCTGTAACAGCCCTTGTAATACTCCAATTTTAGGAGTACAAAAACCTAATGGACAATGGAGGCTGGTGCAAGATCTCAGGCTTATTAATGAAGCTGTGATCCCTTTATACCCAGCTGTACCCAACCCATACACCTTGCTCTCTCAAATACCAGGAGAGGCACAATGGTTTACAGTCCTGGACCTTAAGGATGCCTTTTTCTGTGTTCCATTACATCCTGACTCCCAGTTTCTGTTCGCCTTTGAAGACCCTTTAGACCAGAATTCTCAGCTCACCTGGACAGTGTTACCCCAGGGCTTTCGAGATAGCCCCCACCTGTTTAGCCAGGCTCTAGCTCAGGACCTTAGTCATTTTTCACATCCAGGTACCCTGGTTCTTCAATATATGGATGACTTACTTTTAACTGCTAACTCAGAAACCCAGTGTCAGCAGGCCACCCAGGACCTCCTAAACTTTCTAGCCACCTGTGGATATAAGGTTTCCAAATCAAAAGCCCAACTTTGTAGGCAAGAGGTAAAATACCTAGGGCTTGTCTTGTCTAAGGGCACTCGGGCCCTTGATGGAGAATGTCTCCAACCCATACTGGCCTTCCCCCATCCAAAGACCTTAAAACAGTTGCAGGGATTCTTAGGAATAACTGGTTTCTGCCGATTGTGGATTCCCAGATATGGTGAAATAGCCAAACCCCTATACACTCTGATTAAGGAAACCCAAAAAGCTAACACCCATCTAATACAATGGGACCCTATAGCTGAGGCAGCCTTTCAGACCCTGAAGCAGGCACTACTGCAGGCTCCAGCTCTAAGCCTTCCTACAGGGAACAACTTCTCCCTATATGTTATGGAAAAGTCAGGAGTGGCACTGGGGGTTCTCACCCAGACTTGGGGGACCACGCCACAACCGGTGGCATACCTAAGTAAGGAAATTGATGCAGTAGCAAAAGGCTGGCCACACTGCTTGTGGGTCATAGCCACAGTGGCTATACTGGTCTCAGAAGCTGTTAAAATAGTGCAAGGGAAAGATCTAACTGTATGGACTTCACATGATGTAGCTGGAATTCTGGCCTCTAAAGGAGGCTTATGGCTGTCCGACAACCGCTTACTCAAATATCAGGCCTTAATGCTCGAAGGGCCTGTACTTCAGCTGCGCACATGTGCAGCCCTCAATCCAGCTACTTTTCTTCCTGAGGAAGGGGCAGACAAAGAACATGACTGTCAGCAGGTTATAGCCCAGAACTATGCGGCTCGAGAGGATCTCTTAGAAACTCCTCTAGCCAATCCTGACCTTAACCTATACACTGACGGAAGTTCATTTATGGAAAATGGAGTTCGAAAGGCAGGATATGCAGTAGTCAGTGATCAAACAGTGCTTGAAAGTAATTCCCTTCCTCCTGGAACTAGTGCCCAGCTGGCAGAACTAGTAGCCCTCACTCAAGCTCTAAAATTAGGAAAAGGAAAGAGAATAAACATATACACAGATTCCAAATATGCTTACCTGGTTCTGCATGCTCATGCTGCCATATGGAAGGAAAGAGAGTTCCTAACCTCGGCAGGAACTCCCATTAAATATCATAAGGAAATTTTAGACTTATTACAAGCTGTCCAAGAACCCAAAGAGGTAGCAGTCTTACACTGCCGAGGTCACCAGAAAGGAGATGAGAAGGAAGCAGAGGGCAATCGCCGAGCTGACCTAGAGGCAAAAAGGGCAGCTAGACAAGAATTTATAGAAGGACCTTTAATATGGGAAAACCCTCTCCAGGGAACTAGACCTCAATACTCATCAGAAGAGGTAGAGTGGGCAATCTCCCAGGGACATAATCTTCTTCCTTCAGGATGGCTAGCTACTGAGGAGGGAAAAGTACTCCTACCTGCTGCCAGCCAGTGGAAAGTACTTAAAACCCTACATCAAACCTTCCATATGGGTATAGAGAATACACATCGAATGGCCAAATCTATATTTACAGAAAAAGGTCTCTTCAAGGCTGTCCAACAAATAGTCAAAGGATGTGAAATATGCCAGAAAAATAATCCTTTGGCACATCGCAAAGCTCCTCCTGGGGAACAGCGAACCGGTCATTACCCAGGTGAGGACTGGCAAATGGACTTTACCCATATGCCTAAGTCTCAGGGATATCAATATCTGCTAGTATGTGTAGATACCTACACAAACTGGGTTGAGGCTTTCCCCTGTAGAACAGAAAAAGCCCAAGAGGTAATAAAAGTCCTGATTAATGAAATAATCCCTAGATTCAGACTCCCTCGCTGTCTTCAAAGTGATAACGGCCCTGCCTTCAAAGCTGCAGTGACCCAAGGAATTTCCAAGGCATTAGGAATACAGTATCATCTCCACTGTGCTTGGAGGCCTCAGTCCTCAGGGAAAGTGGAAAAGATGAATGAAATTCTTAAGAGACACCTAAAGAAACTGGCACAGGAAACCCACCTCCCGTGGCCTTCTCTATTACCAATAGCACTCTTAAGGGCTCGAAACTCTCCTCAGAAAATAGGACTCAGCCCCTATGAGATGCTTTATGGATGGCCCTTTCTTACTAATGATCTTGTATTAGACAAGGAAACAGCAGATCTAATAAAAGATATAACCTCCTTGGCTAAGTACCAACAGGCTCTTAGGACCTTACCTGAAGGACACCCTAGGGAAAAAGGGAAAGAGTTGTTCTGCCCTGGAGACTTGGTGCTTGTTAAGTCTCTCCCCTCAGACTCTCCCTCTCTAGGCCCATCTTGGGAAGGACCATTTTCAGTTATTCTGTCTACCCCAATGGCAGTTAAGGTGGTGGGAATTGACTCATGGATTCATCACACTCGGCTGAAGCCTTGGACACCTCCTGAAGAAAATCATAGGCCCTCCACTCCACAGCCTGAAGTTCCAGGAGACGACCCTTCATACACCTGCGAGCCATTAGAGGATCTACATTTGCTCTTCCAACAAGATCCCCAGGAAAGTAACTACTCATGAGTCTAATTTACATCCTGGCATTAACAGGACTATCACTCTTAACTTTGCTATCTGCAACCAGACTCTATGTGGTTGCCCCTACGGATTGGCCCACATCTCATAAACTCTCCTTATCCATGGCTTACTGCCTAATTATACTTCTAATAGCTCTCCTCCTCTCCTTCTGATATTATCACTCCCTTACTATGACCTCTCTTGTGGTCCTCATTATCCTCTTTGCTGCCTGTCCCCTGCTTCACTCCCATCCCACCTGCAAATGCACCCAAAGGGGTTACAACTGGAACAATATTGAACAGGGAACAAGTCCACTCTCTGGTTGGTCTTGGGGATTAGGTCAACAGGCCTCCTTTATATCCTATACTTATATGCACAAAAATTGCTATACATCTGCTTCCCTGTGCACATACAACTCACACACATATTGGACAGGAACAATAACTGGGCCTAATTGTCCAGCTGCTAAGGGAACCACCGCCTGCTGGACATATTTTACCCACATTGGGACTTCAGATGGGGGCAGAGTTCAAGATCAAGGCACAGGAACAGTATCTCCAGAAAGTAATCTCTAGCCTAAGCAAACCACGAAGTACCCCCAGTCCCTACAAGGGCCTGGACCTTTCTAAGCTACATGAGACCCTTTACACACACTCCCATATGCTCAACTTATTTAACACCACCCTCACAGGAATGCATGAGGCCTCCTCTGACAACCCAACTGACTGTTGGATGTGCCTCCCACTATGCTTCAAACCATATATCCCGGTTCCAGTTGCATACTTATGGAACATAACTAACCCACCCTCAAATCATACGGGGCTTATAGGTCCCTTGGTCACCAATCTACAAATTCCATCTACCTCAAACTTCACTTGTGTGAACTCTACTAACAACTCCAATGCTTCTCTATGCCAAAACTGGGTTGCCTTACCCCCAGGCCTCAACTGCCTAGCACCAGGCTTATTCTTTGTTTGTGGCACCTCAGCCTACCACTGCCTAAACACTACCCCTGGGGAAATATGCCTCCTCTCCTTCCTAGTGCCCCCCCCATGACGATTTACACCGACTCTCAGGTGCAGAGTCTTCTCTTGCCAAGGCCACGCCATACTCAAACTCCAATTATTCCTTTCATAGTGTGGGGGGTGGCAGAATAATAGGAGGACTTGGTACTGGCATTGGAAAAGTTACTACCTCTACTCAGTTTTACTATAAGCTATCTCAGGAGTTAAATAATAACATGGAAAAGGTTGCTAATTCTCTCTGCAAAGGCAGCTCACTCTGCAAAAGCAGCTTAACTCTTTAGCTGCCATAGCTCTTCAAAATAGAAGGGCCCTGAATCTGCTAACAGCAGAGAGAGAAGGAACCTGTCTCTTTCTAGGAGAAAAATGCTGCTACTTTGTAAACCAATCAGGTATTGTCTTAAACAAAGTTAAAGAACTCAGGGATCAATCAAACACAGGGCTAAAGACCTTCAAGAAACAGGATCGTGGAGCTTGTTTAACAACTGGCTGTCCTGGCTCTATCCTCTCCTAGGCCCGTTTATAACCATTCTATTATTGCTGGCCTTTGGGCCTCTCATCTTTAACCTTCTTGTCAAATTCGTTTCTTCTAGAATTAAAGCCATAAGGCCACAAATGATCCTGCAAATGGAACCCCGGATGTCCTTGGCTCATGAGTTCTATCGTGGACCTCTGGATCAACCTGCACCCCTGGAAGACTCCCCTCTGGAGGAAACCTCAACTGCAGGGCCCCTTCTATGCCCCAATTCAGCAGGAAGTAGTTAAAGAGCGATCGACGCCCATGTCCCAACAGCAGTTGGACTTTCCTGTTCAGAGGGGGGACTGAGGGACTGTGTTTCTTCCCGGGTCCACAAGGGGCGTGGTTTCAAAGGCAAGAATTTCCCAGCGCCATGCCTCCACTCCTCCATCTGGGAACCTAAAGTTCTTTGTCTAAMTTTCCCGCGCGCGGGCTAGGTAGARAAATCCAGGTGAGGCTCCTTTGTCCCAAAAAGCCCGCCGAAAGCTCTGGGAGGAGGGGGCTCTGTTGTGGACCAAGCAGCCAATCCAGTATCAGAGTCAAAGATCAATCACTCCAGAGGAAGTTTGAAAGAACTCCTCTCATTGGACGAGAACATGAATGGGGAGGGAATAACTCCAGGGTTAAAAGTCCCCCTGCTCCTTACCTATATGGATCTCCATCTCTGGATCCCCTCCCACGACAGCGTGGGAGCTGTCTCTCTCTCTTTCTCTCTCTCTGCCTAATAAATCGCTTTTCTGCAAAACTCTTTGGGTTCATGATATTTACTTGAACGGTAAGCTCACCGCGCCTCCGGGGCCCCTTCCCCCATTCTTGGGGGTRAGAGAGGCCAAGAACCTGGAATTAAGATCGGGGAGCTGGGAGTGCAYCCGACCCCAGTTACA

>ERV1-1_SaiBol_PVconsensus_LTR

TGAGGGACTGTGTTTCTTCCCGGGTCCACAAGGGGCGTGGTTTCAAAGGCAAGAATTTCCCAGCGCCATGCCTCCACTCCTCCATCTGGGAACCTAAAGTTCTTTGTCTAAMTTTCCCGCGCGCGGGCTAGGTAGARAAATCCAGGTGAGGCTCCTTTGTCCCAAAAAGCCCGCCGAAAGCTCTGGGAGGAGGGGGCTCTGTTGTGGACCAAGCAGCCAATCCAGTATCAGAGTCAAAGATCAATCACTCCAGAGGAAGTTTGAAAGAACTCCTCTCATTGGACGAGAACATGAATGGGGAGGGAATAACTCCAGGGTTAAAAGTCCCCCTGCTCCTTACCTATATGGATCTCCATCTCTGGATCCCCTCCCACGACAGCGTGGGAGCTGTCTCTCTCTCTTTCTCTCTCTCTGCCTAATAAATCGCTTTTCTGCAAAACTCTTTGGGTTCATGATATTTACTTGAACGGTAAGCTCACCGCGCCTCCGGGGCCCCTTCCCCCATTCTTGGGGGTRAGAGAGGCCAAGAACCTGGAATTAAGATCGGGGAGCTGGGAGTGCAYCCGACCCCAGTTACA

>ERV1-1_SaiBol_PVconsensus_INTERNAL

TCTTTGGCGAGCCAGCCAGGAGACATCAGACTTCAGAGTGGTGAGTGATATTGGACCCCTTTCACTTGCTAATCTGTCCTTTCTCTTCTTTTAAATTTGTTAAGGAGATAAAGACCGGGCGCCTGTTGGCCATCTAAGAGCAACTAGCGCGGCCACCGGCTAAAGACACGGGTGTCAGGCTTTCTGGGAACGGACTCTCCAGCAACCCCCAGCTTTTCAGAACTGGGAGCGTTGGTCTAGCCCCGAACCAGCTTCCCTTTCTTGCCTGCTCTTTCTTTCTCTGGGCCAAGCCGAGGGCCGGCAGGAGGAAAGGCAATGGTCCTAAACTCCCGGTTCAGTCCAGTTGAGGTTATGGCCCTACTTAGACCTCCTAAGAAAAAGCCGCCTGTGTGAGCCACGCCCAGCCTTTCAGACCCCAACCTCCTGGGTCCAGGCAATTTCCATGAGGCAAACTTCCCGGCTCTTTCCTCTAAGATATTTCCTACACATGAGGCTATTACCCTTCTTGCTAGGACTTTGAGGCTCTTGGGGTGGCAGTCCAGGGACCCTTGCCCGTGGTGCCCTCAGGCTTATGCCTATGAGGACCTCCTACAGTATAAATGCACAGGGCCAGTTCCCTTAGTCCGCAGTGCTAGGTTATGTCCTAACAGAGCCAGAGTCTATCAGTGGAACCTTTTGGGGAAATCCAGGTACTGGAGGCTATAGGACATTGTGTTTTTTGTCACCCGCAATTTCCCAATCCAACTGAATGGTGCCAGAATCTAATGATGTACAATTTGTCTTGCCTAGGATTGGCTCTCCCTACGGCAGAACTTCTCCATCTAGAATACCATAGTCAGACCTGGTCCAAGGCTCAAATAGTTAATTACAGGCCCCACAACCCTAGGGCATATTCCTATAATGGAAAGTGGGGTGGGGGAGGAGGCCCGGGTCCTGAGGGCATTGTTCCAGCCTCAGCCATGTGGCCAGCCATTACCTTCTTTAAGATTGTTGTGCTTAGAGGCCCCTCCCAAGAAGTTTATCCTCCTAGGGCAGTTTTATGATCAGGCCTATGTTAATTGAACTCTTGCTAGGGAATATTGGCACCCATCACCCAAGCCTCACTGGCCTAGTTCTTCAAGGACACGCTGGTACAGGACCACTAATCCCAGCAGTCCCCATGTCCTTATGGTGGTCAATAAGGACATGTAGACAGTGAGGGTGTGCCCATGACGCTGGTAAGCATGATTAAACCCAGCACATGGGAGGGGCCCAGAAGATGATCACGCATCTAGGGCACAAGGAAATTACAAGGTGCTATAACTGGGGGATGCCCCAGGGCTAGCAGTTGCAAGGAATAACCAGGGATGCGGGCATCCCTGTGTTCAATTTCAGATGGGTGCTACTCTTCCCAAGGCAAAATCACCCTTAAAGTGTATCCTGGATAACTGGGACCAATTTGACACACAATCTTTGAAGAAGAAGTGGCTCATTTTTCTCTGCCAAACAGCGTGGCCACAATACCCTCTGCTGAGTGGAGAAACCTGGCCTCCTAAGGGAAGTATAAATTATAACACCCTTCTACAATTAGACCTGTTCTGTAAGAGGGAAGGTAAATGGATTGAGGTCCCCTATGTGCAAGTTTTCTTTTCTCTAAGGGATAATCCTCAGCTATGTAAGGCCTGTAACCTATACCCTACTGGCCCCTCCTCAGGACTACCCCCATATTTAGGACTCCCTGTGGCTCCTCCCTCCACCAATGCTGACCAGGTCTCCTCAGCTCCTATAACCCAAGAGGAGTCAGGGAAGGCCAAAATGGCCAAGGCACCTCAGGCTACTAGGATGCCCCAACTCTGCCCCCTACAGGCAGTAGGAGGAGAATTTGGCCCAACCAAGGTACATACTCCCTTTTCACTTTCTGACTTAAAGCAAATCAAGGCGGACTTAGGAAAATTTTCAGATGACCCAGACAAGTACATAGATGTTCTGCAGGGCCTGGGTCAGTCCTTTGAATTAGATTGGAAAGATATCATGTTATTGCTTAGTCAGACCCTAACCAGCAATGAGAGAGAGGCTGCTCTAGCAGTGGCTCAAGAGTTTGGGGACACCTGGTACCTTAGTCAAGTACATGACCAAATGACGCCAGAGGAAAAGGATAGATTCCCTACAGGTAGACAAGCAGTCCCTAGCATGGACCCTCACTGGGATCCTGACTCAGAACAGGGGGACTGGAGTCGTAGGCACCTATTGACCTGCATACTTGAGGGATTAAGGCGAACTAGGAAAAAGCCCATGAACTATGCAATGTTATCCACCATAACTCAGGGAAAGGAAGAAAACCCAACAGCCTTCCTTGAGTGATTACGGGAGGCCTTAAGAAAGTATACTCCCCTGTCACCAGACTCCATCGAGGGTCAGTTAATACTAAAAGACAAATTTATTATGCAATCAGCAGCAGACATTAGGAGGAAGCTCCAGAAGCTGGCCCTGGGGCCAGAACAGAGTCTAGAATCATTACTAAATCTAGCAACCTCGGTGTTTTATAATAGAGACCAGGAGGAACAGGCTGAAAGGAACAGGAGAGACCAGAGAAAAGCCGCAGCCCTAGTCATGGCCTTCAGGCAAGCAGACCCAAGGGGCTCAGAAGAGGGAAAGGCTTGGGTTAGCCGCCAGTCTGGCAGAGCTTGCTACCACTGCGGCCTAACGGGACACTTTAAAAGAAATTGTCCCCAGAGGAATGGACCACCTCCTCGTCCTTGCCCACTGTGCCAAGGGGATCACTGGAAAGCACACTGTCCCAGGGGATGAAGGTTCCCAGGGCCTGAGGTGGCTAACCAGATGACCCAACAGCAGGACTGAGGGTGCCCGGGGCAAGTGCCAGCACAAGCCATCACCCTCACCGAGCCCCGGGTAAAGCTAACCATAGAGGGCCAGGAGGTTGATCTTCTCCTGGACACTGGCATGGCCTTCTCAGTTTTGCTTTCCTGTCCTGGACAGTTGTCCTCCAAGTCTGTAACTGTCCGAGGAGTCCTAGGACAAGCAGTCTCCCGATATTTCTCCCAGCCCCTAAGCTGTAACTGGGGAGATATACTCTTCTCCCATGCCTTCCTCATCATGCCAGAAAGTCCCACTCCCTTACTAGGGCGGGACCTGTTAGCCAGAGCAGGAGCCATTATCTATATGAACATCATCAGGGAGGAAATCCCTCTCTGCTGCCCATTATTTAAAGAAGGGATTAATCCTGAGGTTTGGGCAATGGAGGGACAATTTGGATGGGCAAAGAATGCATGCCCAATTCGAATAAGGCTAAAGGATCCCACCTCTTTTCCTTACCAAAGACAGTACCCCCTAAGACCGGAAGCCAAAAAGGGGCTACAGGATATTGTAAGGAACTTAAAAGCTCAGGGTCTAGTAAAGTCCTGTAACAGCCCTTGTAATACTCCAATTTTAGGAGTACAAAAACCTAATGGACAATGGAGGCTGGTGCAAGATCTCAGGCTTATTAATGAAGCTGTGATCCCTTTATACCCAGCTGTACCCAACCCATACACCTTGCTCTCTCAAATACCAGGAGAGGCACAATGGTTTACAGTCCTGGACCTTAAGGATGCCTTTTTCTGTGTTCCATTACATCCTGACTCCCAGTTTCTGTTCGCCTTTGAAGACCCTTTAGACCAGAATTCTCAGCTCACCTGGACAGTGTTACCCCAGGGCTTTCGAGATAGCCCCCACCTGTTTAGCCAGGCTCTAGCTCAGGACCTTAGTCATTTTTCACATCCAGGTACCCTGGTTCTTCAATATATGGATGACTTACTTTTAACTGCTAACTCAGAAACCCAGTGTCAGCAGGCCACCCAGGACCTCCTAAACTTTCTAGCCACCTGTGGATATAAGGTTTCCAAATCAAAAGCCCAACTTTGTAGGCAAGAGGTAAAATACCTAGGGCTTGTCTTGTCTAAGGGCACTCGGGCCCTTGATGGAGAATGTCTCCAACCCATACTGGCCTTCCCCCATCCAAAGACCTTAAAACAGTTGCAGGGATTCTTAGGAATAACTGGTTTCTGCCGATTGTGGATTCCCAGATATGGTGAAATAGCCAAACCCCTATACACTCTGATTAAGGAAACCCAAAAAGCTAACACCCATCTAATACAATGGGACCCTATAGCTGAGGCAGCCTTTCAGACCCTGAAGCAGGCACTACTGCAGGCTCCAGCTCTAAGCCTTCCTACAGGGAACAACTTCTCCCTATATGTTATGGAAAAGTCAGGAGTGGCACTGGGGGTTCTCACCCAGACTTGGGGGACCACGCCACAACCGGTGGCATACCTAAGTAAGGAAATTGATGCAGTAGCAAAAGGCTGGCCACACTGCTTGTGGGTCATAGCCACAGTGGCTATACTGGTCTCAGAAGCTGTTAAAATAGTGCAAGGGAAAGATCTAACTGTATGGACTTCACATGATGTAGCTGGAATTCTGGCCTCTAAAGGAGGCTTATGGCTGTCCGACAACCGCTTACTCAAATATCAGGCCTTAATGCTCGAAGGGCCTGTACTTCAGCTGCGCACATGTGCAGCCCTCAATCCAGCTACTTTTCTTCCTGAGGAAGGGGCAGACAAAGAACATGACTGTCAGCAGGTTATAGCCCAGAACTATGCGGCTCGAGAGGATCTCTTAGAAACTCCTCTAGCCAATCCTGACCTTAACCTATACACTGACGGAAGTTCATTTATGGAAAATGGAGTTCGAAAGGCAGGATATGCAGTAGTCAGTGATCAAACAGTGCTTGAAAGTAATTCCCTTCCTCCTGGAACTAGTGCCCAGCTGGCAGAACTAGTAGCCCTCACTCAAGCTCTAAAATTAGGAAAAGGAAAGAGAATAAACATATACACAGATTCCAAATATGCTTACCTGGTTCTGCATGCTCATGCTGCCATATGGAAGGAAAGAGAGTTCCTAACCTCGGCAGGAACTCCCATTAAATATCATAAGGAAATTTTAGACTTATTACAAGCTGTCCAAGAACCCAAAGAGGTAGCAGTCTTACACTGCCGAGGTCACCAGAAAGGAGATGAGAAGGAAGCAGAGGGCAATCGCCGAGCTGACCTAGAGGCAAAAAGGGCAGCTAGACAAGAATTTATAGAAGGACCTTTAATATGGGAAAACCCTCTCCAGGGAACTAGACCTCAATACTCATCAGAAGAGGTAGAGTGGGCAATCTCCCAGGGACATAATCTTCTTCCTTCAGGATGGCTAGCTACTGAGGAGGGAAAAGTACTCCTACCTGCTGCCAGCCAGTGGAAAGTACTTAAAACCCTACATCAAACCTTCCATATGGGTATAGAGAATACACATCGAATGGCCAAATCTATATTTACAGAAAAAGGTCTCTTCAAGGCTGTCCAACAAATAGTCAAAGGATGTGAAATATGCCAGAAAAATAATCCTTTGGCACATCGCAAAGCTCCTCCTGGGGAACAGCGAACCGGTCATTACCCAGGTGAGGACTGGCAAATGGACTTTACCCATATGCCTAAGTCTCAGGGATATCAATATCTGCTAGTATGTGTAGATACCTACACAAACTGGGTTGAGGCTTTCCCCTGTAGAACAGAAAAAGCCCAAGAGGTAATAAAAGTCCTGATTAATGAAATAATCCCTAGATTCAGACTCCCTCGCTGTCTTCAAAGTGATAACGGCCCTGCCTTCAAAGCTGCAGTGACCCAAGGAATTTCCAAGGCATTAGGAATACAGTATCATCTCCACTGTGCTTGGAGGCCTCAGTCCTCAGGGAAAGTGGAAAAGATGAATGAAATTCTTAAGAGACACCTAAAGAAACTGGCACAGGAAACCCACCTCCCGTGGCCTTCTCTATTACCAATAGCACTCTTAAGGGCTCGAAACTCTCCTCAGAAAATAGGACTCAGCCCCTATGAGATGCTTTATGGATGGCCCTTTCTTACTAATGATCTTGTATTAGACAAGGAAACAGCAGATCTAATAAAAGATATAACCTCCTTGGCTAAGTACCAACAGGCTCTTAGGACCTTACCTGAAGGACACCCTAGGGAAAAAGGGAAAGAGTTGTTCTGCCCTGGAGACTTGGTGCTTGTTAAGTCTCTCCCCTCAGACTCTCCCTCTCTAGGCCCATCTTGGGAAGGACCATTTTCAGTTATTCTGTCTACCCCAATGGCAGTTAAGGTGGTGGGAATTGACTCATGGATTCATCACACTCGGCTGAAGCCTTGGACACCTCCTGAAGAAAATCATAGGCCCTCCACTCCACAGCCTGAAGTTCCAGGAGACGACCCTTCATACACCTGCGAGCCATTAGAGGATCTACATTTGCTCTTCCAACAAGATCCCCAGGAAAGTAACTACTCATGAGTCTAATTTACATCCTGGCATTAACAGGACTATCACTCTTAACTTTGCTATCTGCAACCAGACTCTATGTGGTTGCCCCTACGGATTGGCCCACATCTCATAAACTCTCCTTATCCATGGCTTACTGCCTAATTATACTTCTAATAGCTCTCCTCCTCTCCTTCTGATATTATCACTCCCTTACTATGACCTCTCTTGTGGTCCTCATTATCCTCTTTGCTGCCTGTCCCCTGCTTCACTCCCATCCCACCTGCAAATGCACCCAAAGGGGTTACAACTGGAACAATATTGAACAGGGAACAAGTCCACTCTCTGGTTGGTCTTGGGGATTAGGTCAACAGGCCTCCTTTATATCCTATACTTATATGCACAAAAATTGCTATACATCTGCTTCCCTGTGCACATACAACTCACACACATATTGGACAGGAACAATAACTGGGCCTAATTGTCCAGCTGCTAAGGGAACCACCGCCTGCTGGACATATTTTACCCACATTGGGACTTCAGATGGGGGCAGAGTTCAAGATCAAGGCACAGGAACAGTATCTCCAGAAAGTAATCTCTAGCCTAAGCAAACCACGAAGTACCCCCAGTCCCTACAAGGGCCTGGACCTTTCTAAGCTACATGAGACCCTTTACACACACTCCCATATGCTCAACTTATTTAACACCACCCTCACAGGAATGCATGAGGCCTCCTCTGACAACCCAACTGACTGTTGGATGTGCCTCCCACTATGCTTCAAACCATATATCCCGGTTCCAGTTGCATACTTATGGAACATAACTAACCCACCCTCAAATCATACGGGGCTTATAGGTCCCTTGGTCACCAATCTACAAATTCCATCTACCTCAAACTTCACTTGTGTGAACTCTACTAACAACTCCAATGCTTCTCTATGCCAAAACTGGGTTGCCTTACCCCCAGGCCTCAACTGCCTAGCACCAGGCTTATTCTTTGTTTGTGGCACCTCAGCCTACCACTGCCTAAACACTACCCCTGGGGAAATATGCCTCCTCTCCTTCCTAGTGCCCCCCCCATGACGATTTACACCGACTCTCAGGTGCAGAGTCTTCTCTTGCCAAGGCCACGCCATACTCAAACTCCAATTATTCCTTTCATAGTGTGGGGGGTGGCAGAATAATAGGAGGACTTGGTACTGGCATTGGAAAAGTTACTACCTCTACTCAGTTTTACTATAAGCTATCTCAGGAGTTAAATAATAACATGGAAAAGGTTGCTAATTCTCTCTGCAAAGGCAGCTCACTCTGCAAAAGCAGCTTAACTCTTTAGCTGCCATAGCTCTTCAAAATAGAAGGGCCCTGAATCTGCTAACAGCAGAGAGAGAAGGAACCTGTCTCTTTCTAGGAGAAAAATGCTGCTACTTTGTAAACCAATCAGGTATTGTCTTAAACAAAGTTAAAGAACTCAGGGATCAATCAAACACAGGGCTAAAGACCTTCAAGAAACAGGATCGTGGAGCTTGTTTAACAACTGGCTGTCCTGGCTCTATCCTCTCCTAGGCCCGTTTATAACCATTCTATTATTGCTGGCCTTTGGGCCTCTCATCTTTAACCTTCTTGTCAAATTCGTTTCTTCTAGAATTAAAGCCATAAGGCCACAAATGATCCTGCAAATGGAACCCCGGATGTCCTTGGCTCATGAGTTCTATCGTGGACCTCTGGATCAACCTGCACCCCTGGAAGACTCCCCTCTGGAGGAAACCTCAACTGCAGGGCCCCTTCTATGCCCCAATTCAGCAGGAAGTAGTTAAAGAGCGATCGACGCCCATGTCCCAACAGCAGTTGGACTTTCCTGTTCAGAGGGGGGAC
