# Supplementary figures and images for: HERV-W group evolutionary history in non-human primates: characterization of ERV-W orthologs in Catarrhini and related ERV groups in Platyrrhini
Source: BMC Evol Biol. 2018 Jan 19;18:6. doi: 10.1186/s12862-018-1125-1 (PMC5775608; doi:10.1186/s12862-018-1125-1)

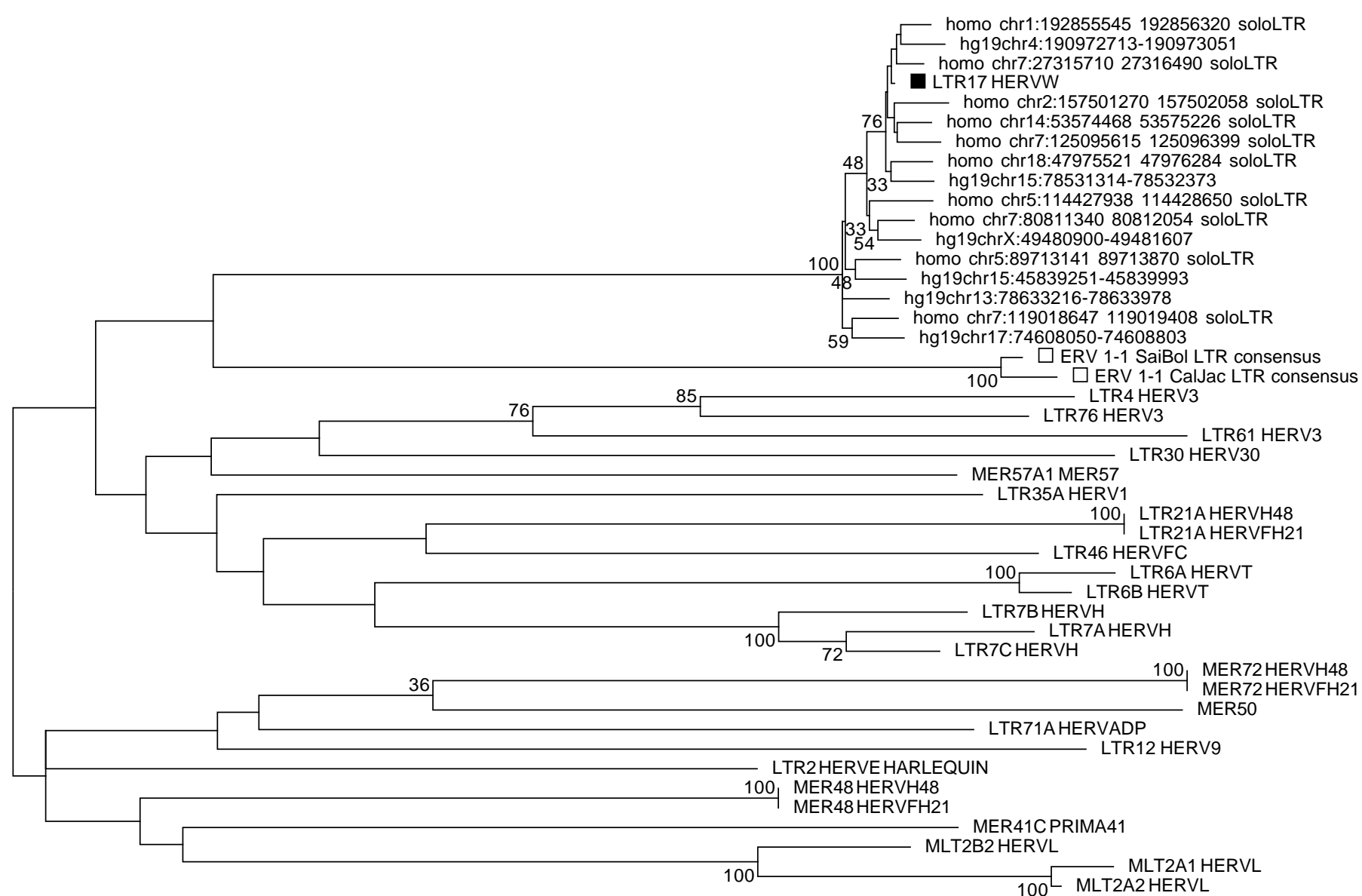

0.2

Supplement: Supplementary file 3 — Phylogenetic analysis of human solitary LTRs orthologous to ERV-W loci formed in Rhesus or Gibbon. Gammaretrovirus-like HERV LTR sequences were retrieved from RepBase: the HERV-W group LTR17 reference sequence is marked with a filled square. The ERV1–1 LTR consensus were generated from the Marmoset (CalJac) and Squirrel Monkey (SaiBol) proviral sequence datasets, and are marked with empty squares. Evolutionary relationships were inferred by using the ML method and the Kimura-2-parameter model. The resulting phylogeny was tested using the bootstrap method with 100 replicates: the obtained bootstrap values are reported near each node (bootstrap values lower than 30% are not shown). Length of branches indicates the number of substitutions per site. (PDF 15 kb) [file 12862_2018_1125_MOESM3_ESM.pdf]

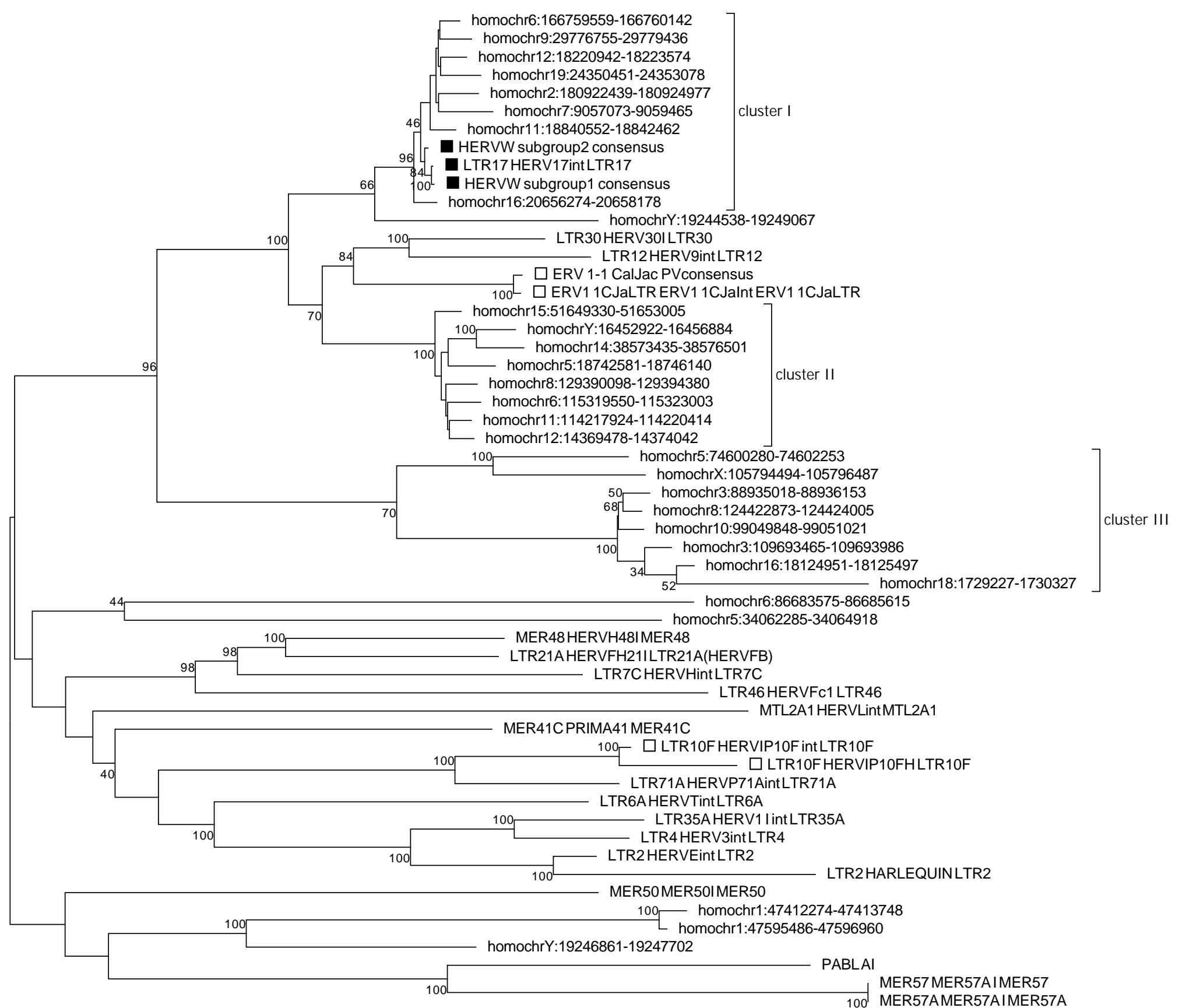

0.2

Supplement: Supplementary file 4 — Phylogenetic analysis of HERV-W-like nucleotide sequences orthologous to ERV-W loci identified in non-human primates by HERV17 BLAT searches. Gammaretrovirus-like HERV reference sequences were retrieved from RepBase. The HERV-W group RepBase LTR17 HERV17 LTR17 reference sequence and the proviral HERV-W subgroup 1 and 2 consensus sequences generated previously [44] are marked with a filled square. The ERV1–1 reference sequence from RepBase and the consensus generated from the proviral sequence dataset in this study are marked with an empty square. Evolutionary relationships were inferred by using the ML method and the Kimura-2-parameter model. The resulting phylogeny was tested using the bootstrap method with 100 replicates: bootstrap values are reported near each node (bootstrap values lower than 30% are not shown). Length of branches indicates the number of substitutions per site. (PDF 20 kb) [file 12862_2018_1125_MOESM4_ESM.pdf]

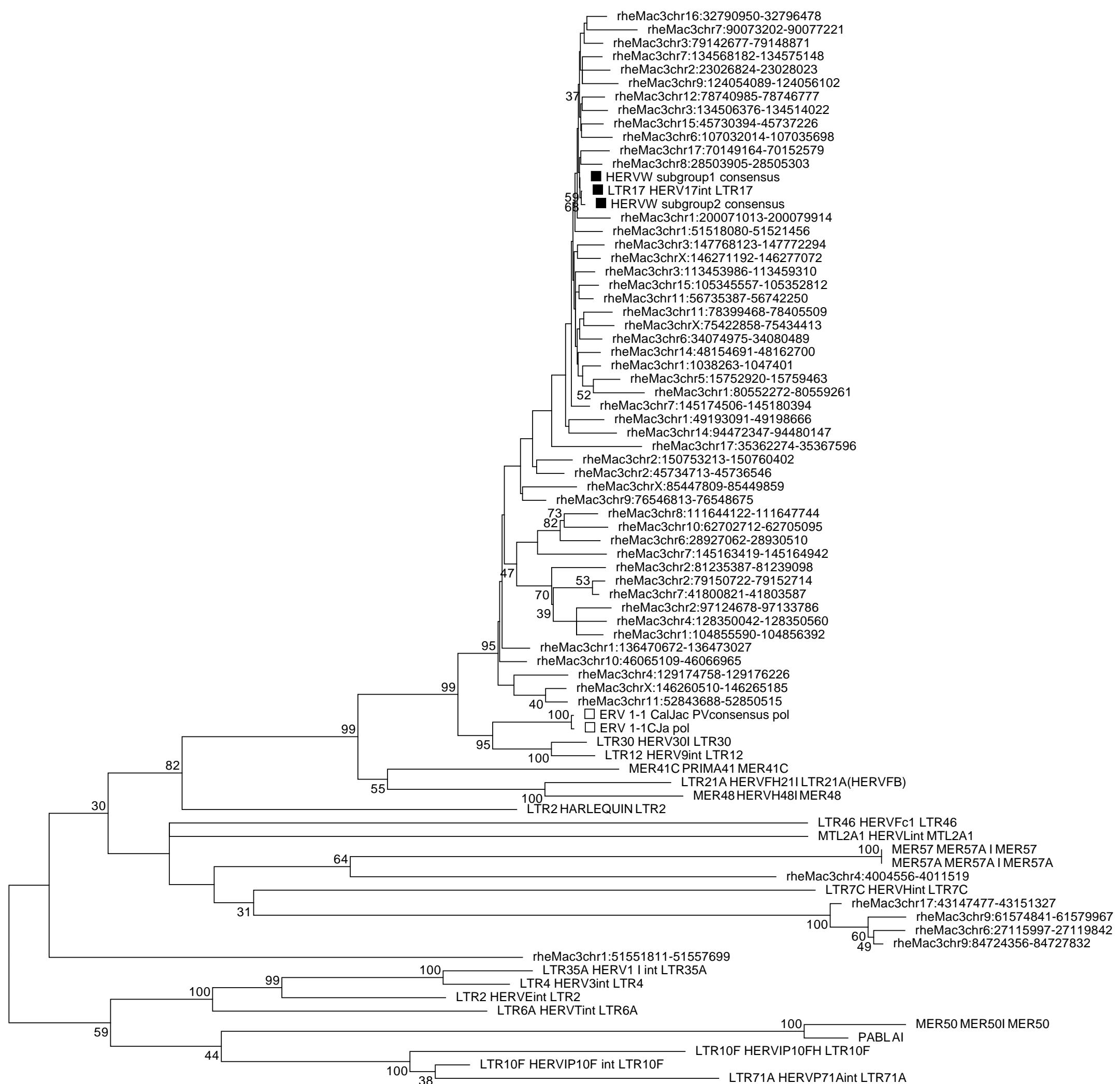

0.2

Supplement: Supplementary file 5 — Phylogenetic analysis of pol gene nucleotide sequence from Rhesus ERV-W loci lacking an ortholog in the human reference genome. Gammaretrovirus-like HERV pol gene reference sequences were retrieved from RepBase. The HERV-W group pol sequences from RepBase reference sequence and the proviral HERV-W consensus sequence generated previously [44] are marked with a filled square. The ERV1–1 pol sequences from RepBase reference sequence and the consensus generated from the ERV1–1 sequences dataset in this study are marked with an empty square. Evolutionary relationships were inferred by using the ML method and the Kimura-2-parameter model. The resulting phylogeny was tested using the bootstrap method with 100 replicates: bootstrap values are reported near each node (bootstrap values lower than 30% are not shown). Length of branches indicates the number of substitutions per site. (PDF 23 kb) [file 12862_2018_1125_MOESM5_ESM.pdf]

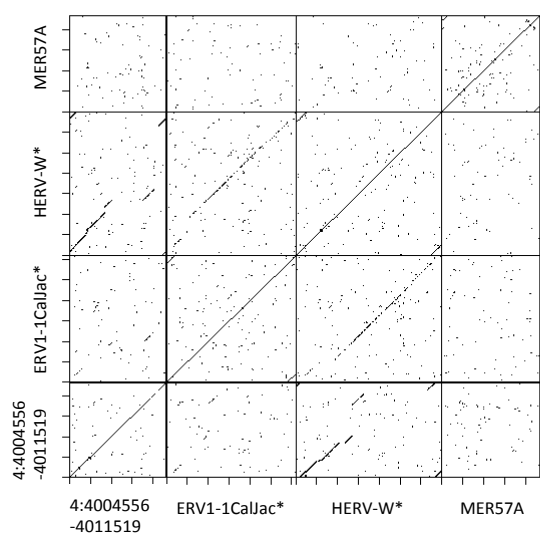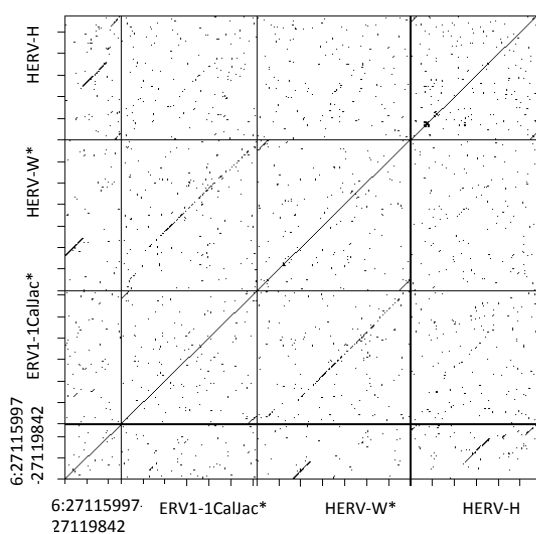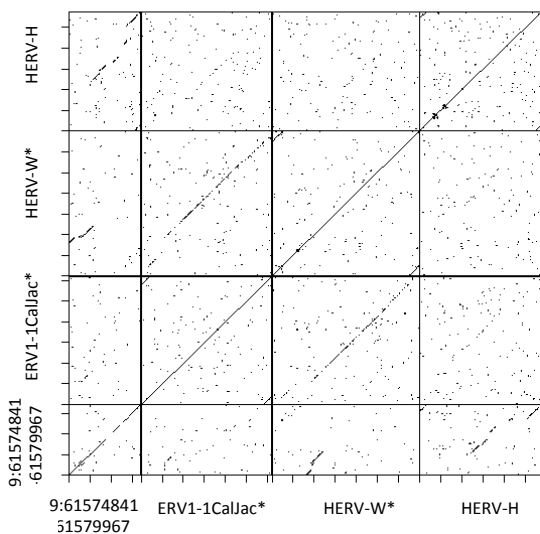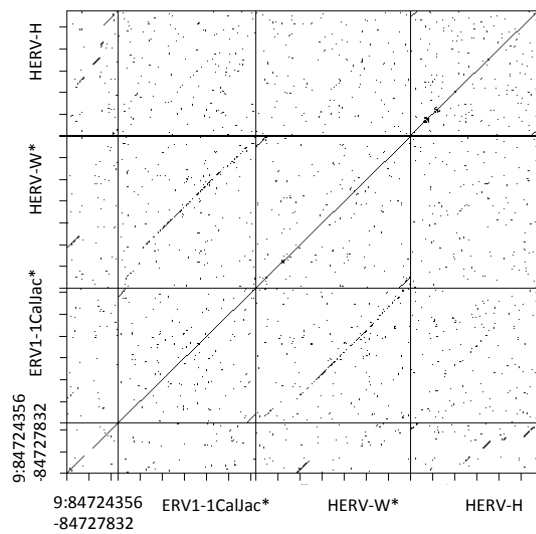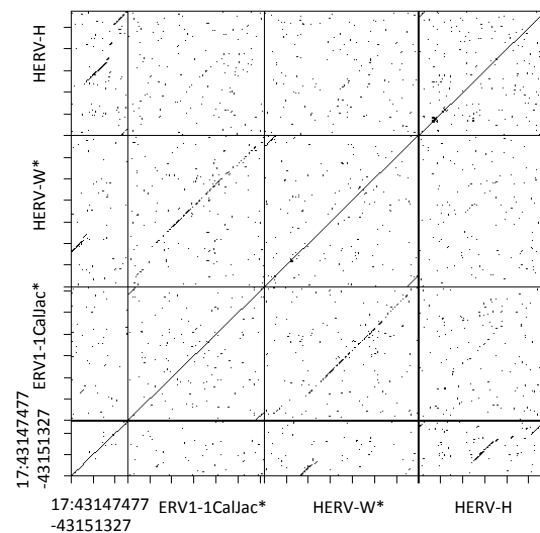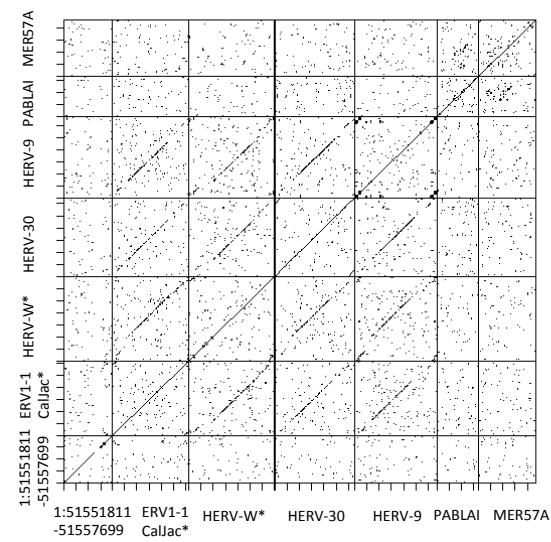

Supplement: Supplementary file 6 — Polydot pairwise analyses of the 6 Rhesus ERV-W nucleotide sequences lacking an ortholog in the human reference genome sequence and showing unclear sequence relationships with other HERV sequences. Analyzed consensus sequences marked “*” were generated in this study. Other sequences were retrieved from RepBase. (PDF 174 kb) [file 12862_2018_1125_MOESM6_ESM.pdf]
